# Aerobic Photodegradation of Pyrene-Based Metal–Organic Framework NU-1000 to Terephthalic Acid

**DOI:** 10.1021/acs.chemmater.5c00520

**Published:** 2025-05-02

**Authors:** Wenyi Zeng, Tristan T. Y. Tan, Youven Benseghir, Michael R. Reithofer, Jia Min Chin, Jason Y. C. Lim

**Affiliations:** † 208718 Institute of Materials Research and Engineering (IMRE), Agency for Science, Technology and Research (A*STAR), 2 Fusionopolis Way, Innovis #08-03, Singapore 138634, Republic of Singapore; ‡ Institute of Inorganic Chemistry, Faculty of Chemistry, 27258 University of Vienna, Währinger Str. 42, Vienna 1090, Austria; § Institute of Functional Materials and Catalysis, Faculty of Chemistry, 31285 University of Vienna, Währinger Str. 42, Vienna 1090, Austria; ∥ Department of Materials Science and Engineering, National University of Singapore (NUS), 9 Engineering Drive, Singapore 117576, Republic of Singapore

## Abstract

NU-1000, a pyrene-containing benchmark metal–organic framework (MOF), is well-known for its utility and potential across a wide range of applications. Although the extended π-systems in NU-1000 confer favorable properties for diverse photochemical applications, they also increase the susceptibility of the MOF to photodegradation. However, the photostability of NU-1000 has yet to be systematically studied and remains poorly understood. Herein, we report that in the presence of oxygen, water, and light of appropriate energy, extensive oxidation of the pyrene linkers of NU-1000 can occur to yield terephthalic acid as the major decomposition product. Through extensive mechanistic studies, we show that the open framework structure of NU-1000 greatly facilitates linker oxidation, with more than 25-fold greater linker decomposition from the MOF within 3 h of photoirradiation compared to a homogeneous solution of the linker, brought about by rapid generation of pyrene-centred holes. By determining the specific conditions under which the MOF remains stable, our findings offer not just valuable strategies for preserving the integrity of NU-1000 for controlled applications but also reveal its ability to decompose into benign products under ambient light and oxygen, which could provide an eco-friendly route for avoiding environmental accumulation of MOF materials for various applications.

## Graphical abstract

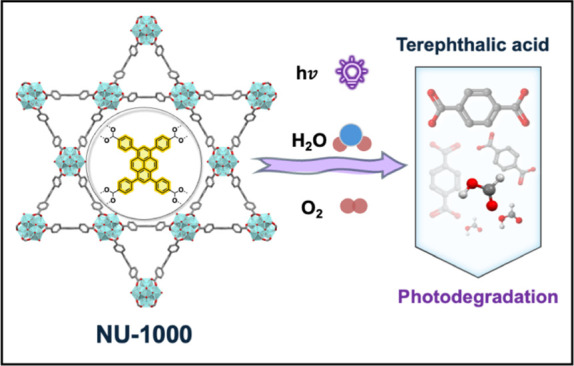

## Introduction

NU-1000, a Zr_6_-based metal organic framework (MOF) first reported by Northwestern University, comprises of [Zr_6_(μ_3_–OH)_4_(μ_3_-O)_4_(OH)_4_(OH_2_)_4_]^8+^ nodes and tetratopic pyrene-based linkers [4,4′,4″,4‴-(pyrene-1,3,6,8-tetrayl)­tetrabenzoic acid (H_4_TBAPy)].^1^ Among pyrene-based MOFs, NU-1000 stands out as an archetypical material due to its excellent thermal, chemical and hydrolytic stability, high internal surface area, large pore size and volume (1.46 cm^3^ g^–1^),^2^ which facilitate efficient substrate diffusion through the mesoporous channels. These desirable features have led to NU-1000 and its derivatives finding diverse applications such as gas capture and separation,^3–8^ drug encapsulation and delivery,^9,10^ energy storage^11^ and adsorption of organic compounds.^12^ Additionally, with its coordinatively unsaturated Zr_6_-oxo nodes, which provide ample sites for Lewis acid catalysis and coordination of different metal cations, NU-1000 is particularly useful for catalysis^13–16^ and remediation of anionic pollutants.^17–19^

The presence of polycyclic aromatic pyrene units, possessing high fluorescence quantum yield and strong absorption in the ultraviolet–visible (UV–vis) region,^2,20^ within the rigid porous structure of MOFs makes the material especially useful for photochemical applications. The conjugated π-system of pyrene contributes to a prolonged excited-state lifetime and efficient electron–hole pair separation—properties which make NU-1000 exceptionally useful as photosensitizer.^2^ For instance, NU-1000 has been demonstrated to be highly effective in the photodegradation of sulfur mustard,^21,22^ as well as the oxidation of amines^23^ and alcohols,^24^ and radical additions of perfluoroalkyl iodides to olefins^25^ (Figure 1).

**Figure 1:**
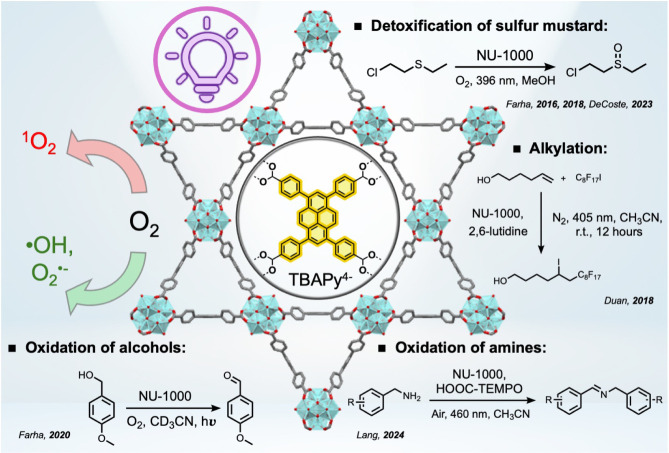
Illustration of the crystal structure of NU-1000, generated from data reported in reference^1^ and chemical structure of the TBAPy^4–^ linker, as well as selected photoreactions facilitated by NU-1000.^21–26^ Singlet oxygen (^1^O_2_), hydroxyl radical (•OH) and superoxide (O_2_^•–^) are known to be generated by NU-1000 upon photoirradiation under aerobic conditions.

Moreover, NU-1000 can be further functionalized postsynthetically for a large range of photocatalytic applications. This includes the incorporation of fullerenes^27^ or BODIPY^28^ for enhanced sulfur mustard detoxification, Re and Co complexes for syngas generation,^29^ polyoxometalates for CO_2_ photoreduction,^30^ encapsulating bismuth oxyiodine (BiOI) for hydrogen evolution reaction^31^ or binary composite Cd_6_Se_6_ cluster, molybdenum sulfide (MoSx) for water splitting reaction.^32,33^ In all cases, the MOF’s photostability is essential for maintaining its catalytic performance and reusability. Of late, in García and coworkers’ exploration of terephthalate MOFs’ photostability,^34^ these materials were shown to undergo photodecarboxylation over weeks while still retaining their crystalline structures in X-ray diffraction (XRD) characterization. However, despite the potential for H_4_TBAPy’s greater photoreactivity with its larger π-system, the photostability of NU-1000 has yet to be elucidated. This is particularly surprising given the well-known photodegradation of pyrene on solid supports such as Al_2_O_3_,^35^ SiO_2_ and montmorillonite.^36^ Additionally, in our previous confocal microscopy experiments photobleaching of NU-1000 has been observed upon prolonged exposure to UV–vis light,^37,38^ hinting at possible photoinstability. This prompted us to further probe the photostability of NU-1000 and the resulting photochemical transformations under irradiation.

We herein report the discovery that NU-1000 can undergo substantial photodegradation in the presence of oxygen, water and light of appropriate energy, leading to the unexpected formation of 1,4-benzenedicarboxylic acid (H_2_BDC, also known as terephthalic acid) as the major degradation product. This finding is particularly remarkable, as it suggests extensive bond cleavage within the pyrene core. In the presence of O_2_ as a mild oxidant, the open framework structure of NU-1000 was found to accelerate the generation of reactive oxygen species compared to its pure ligand, H_4_TBAPy, under photoirradiation. Through this study, we aim to provide insights into the induced degradation of NU-1000 and highlight conditions for extending its functional lifespan. These findings, together with the discovery of its degradation pathway to relatively benign products of terephthalic and formic acids, may provide avenues for designing MOFs with controlled degradation profiles, which may find importance in diverse industrial and environmental applications.

## Experimental Section

### Synthesis of NU-1000

The synthetic approach was adapted from literature.^39^ ZrOCl_2_·8H_2_O (61.25 mg) and benzoic acid (1.25 g) were dissolved in DMF (5 mL) via sonication for 10 min. The reaction solution was placed in a preheated oven (100 °C) for 3 h to form the Zr-oxo cluster. After cooling, trifluoracetic acid (TFA, 25 μL) and H_4_TBAPy (25 mg) were added to the mixture. The resulting yellow mixture was sonicated for 20 min and heated at 100 °C for 18 h. After cooling to room temperature, the light-yellow precipitate was collected by centrifugation and washed twice with DMF. Activation of NU-1000 was performed as reported.^40^ The as-synthesized NU-1000 (ca. 50 mg) was separated from the crude mixture via centrifugation and solvent-exchanged with DMSO (3 × 10 mL). The yellow precipitate was then dispersed in DMSO (15 mL) and HCl (8 M, 0.6 mL), followed by incubation at room temperature for 18 h. The yellow solid was subsequently collected, washed with ethanol (4 × 10 mL), and kept in 10 mL ethanol overnight. To the ethanol suspension, triethylamine (TEA, 5.6 μL) was added and soaked at room temperature for 18 h. After centrifugation and sequential washing with ethanol, acetone, the solid was dried under vacuum at 80 °C for 1 h and then activated at 120 °C for 18 h to yield the NU-1000 as a yellow powder.

### Synthesis of NU-901

The synthetic approach was adapted from literature.^41^ Zr­(acac)_4_ (97 mg) and 4-aminobenzoic acid (3.02 g) were dissolved in DMF (8 mL) via sonication for 10 min. The reaction solution was placed in a preheated oven (80 °C) for 1 h to form the Zr-oxo cluster. After cooling, H_4_TBAPy (40 mg) was added to the mixture. The resulting yellow mixture was sonicated for 20 min and heated at 100 °C for 18 h. After cooling to room temperature, the light-yellow precipitate was separated via centrifugation and washed with DMF twice. The solid was then dispersed in DMF (12 mL) and HCl (8 M, 0.5 mL), followed by heating at 100 °C for 18 h. Afterward, the product was collected, washed with DMF and acetone three times. The solid was first dried under vacuum at 80 °C for 1 h and then activated at 120 °C for 18 h to yield the NU-901 as a yellow powder.

### Photodegradation Experiments

In a 20 mL vial, activated NU-1000 (20 mg, 9.19 μmol) was suspended in a mixture of acetonitrile (5 mL) and deionized (DI) water (2.5 μL). The reaction mixture was irradiated and stirred under 390 nm (52 W) at 33 °C (temperature controlled by an external fan and measured as previously reported),^42^ under an O_2_ atmosphere (provided by an O_2_ filled balloon connected to a syringe, which was pierced through a septum on the reaction vial). The sample vial was placed at about 10 cm in front of the light source. After reaction, the solid residue was collected via centrifugation and dried at 80 °C. For NMR analysis, the dried powder was digested in a mixture of D_2_SO_4_ (25 μL) and *d*_6_-DMSO (500 μL), forming a clear solution. For experiments using LEDs with different wavelengths (e.g., 427 nm–45 W, 440 nm–45 W, 525 nm–44 W), all reaction parameters were kept constant, except for the wavelength and power of the LED light source.

## Results and Discussion

### Synthesis and Characterization of NU-1000

The pyrene-based linker H_4_TBAPy and MOF NU-1000, formulated as [Zr_6_(μ_3_–OH)_4_(μ_3_-O)_4_(OH)_4_(OH_2_)_4_]­(TBAPy)_2_] were synthesized in DMF as previously reported, using benzoic acid as modulator (see Experimental Section). To completely remove benzoic acid and DMF from the MOF sample, in order to avoid peak overlaps during subsequent NMR analyses of NU-1000 degradation (vide infra) with the aromatic protons of TBAPy, the as-synthesized MOF sample was further activated in a mixture of HCl/DMSO solution overnight, then washed with ethanol, and treated with triethylamine in ethanol.^40^ The complete removal of DMF and benzoic acid was confirmed via ^1^H NMR of the digested MOF sample (Figure 2a). Notably, the spectra also showed the unambiguous absence of formates coordinated to the Zr_6_ nodes. The morphology and crystallinity of activated NU-1000 were examined using scanning electron microscopy (SEM) and powder X-ray diffraction (XRD), respectively. From the SEM image shown in Figure 2b, the MOF particle features a rod-shape with an average length around 1 μm. The sample exhibits good crystallinity, as the diffraction pattern was in good agreement with the reported single crystal data,^43^ indicating the high purity and crystallinity of the synthesized sample (Figures 2c and S3).

**Figure 2:**
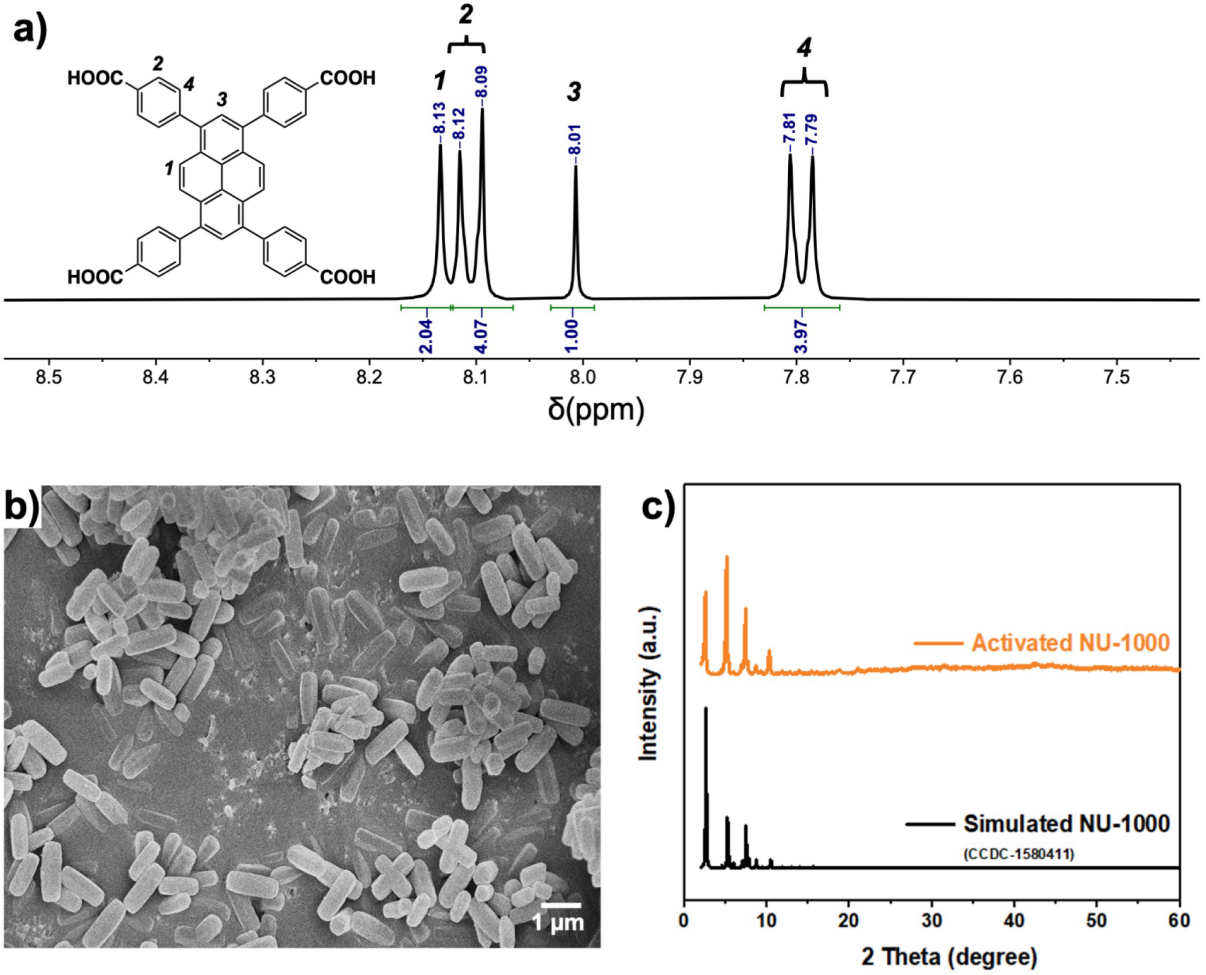
(a) ^1^H NMR of the activated NU-1000 dissolved in D_2_SO_4_/DMSO, showing the sole presence of H_4_TBAPy with benzoic acid and DMF completely removed; (b) SEM images of NU-1000 with 1 μm scale bar; (c) XRD patterns of the activated NU-1000 and the comparison to the reported single crystal data (CCDC-1580411).^39^

### Photooxidation of NU-1000

Our initial assessment of the photostability of NU-1000 under aerobic conditions was conducted using a suspension of activated NU-1000 in acetonitrile (MeCN)/water mixture and irradiation with a 390 nm light emitting diode (LED) array overnight under O_2_ (Figure 3a). After the reaction, the solid displayed a color change from yellow to white. For NMR characterization the collected solid was dissolved in a mixture of D_2_SO_4_/DMSO-*d*_6_, forming a clear solution. As shown in Figure 3b, the signals from the H_4_TBAPy linker molecule disappeared in the irradiated MOF, concomitant with the formation of new products, indicating complete decomposition of the pyrene core within the NU-1000. Characterization of the degradation product by NMR spectroscopy and electrospray ionization mass spectrometry (ESI-MS) unambiguously identified it as 1,4-benzenedicarboxylic acid (H_2_BDC), with the molecular ion peak identified at *m*/*z* = 165.0189 (theoretical *m*/*z* = 165.0193 [M–H]^−^) (see Figures 3c and S15). The signal of the proton of H_2_BDC was detected as a singlet at δ = 8.04 ppm in the ^1^H NMR spectrum, and ^13^C signals of H_2_BDC were detected at δ = 166.9, 134.6, and 129.6 ppm in the ^13^C­{^1^H} NMR spectrum. The ^13^C signals were unambiguously assigned with the aid of ^1^H–^13^C HMBC and ^1^H–^13^C HSQC NMR experiments (see Figure S14). In addition, a small amount of formic acid was detected in both the ^1^H NMR and ^13^C NMR spectra (δ = 8.12 and δ = 163.3 ppm, respectively), which was not originally present in the MOF sample (Figure 2a). No other compounds were present in detectable quantities by NMR, either from the digested solids or the MeCN supernatant.

**Figure 3:**
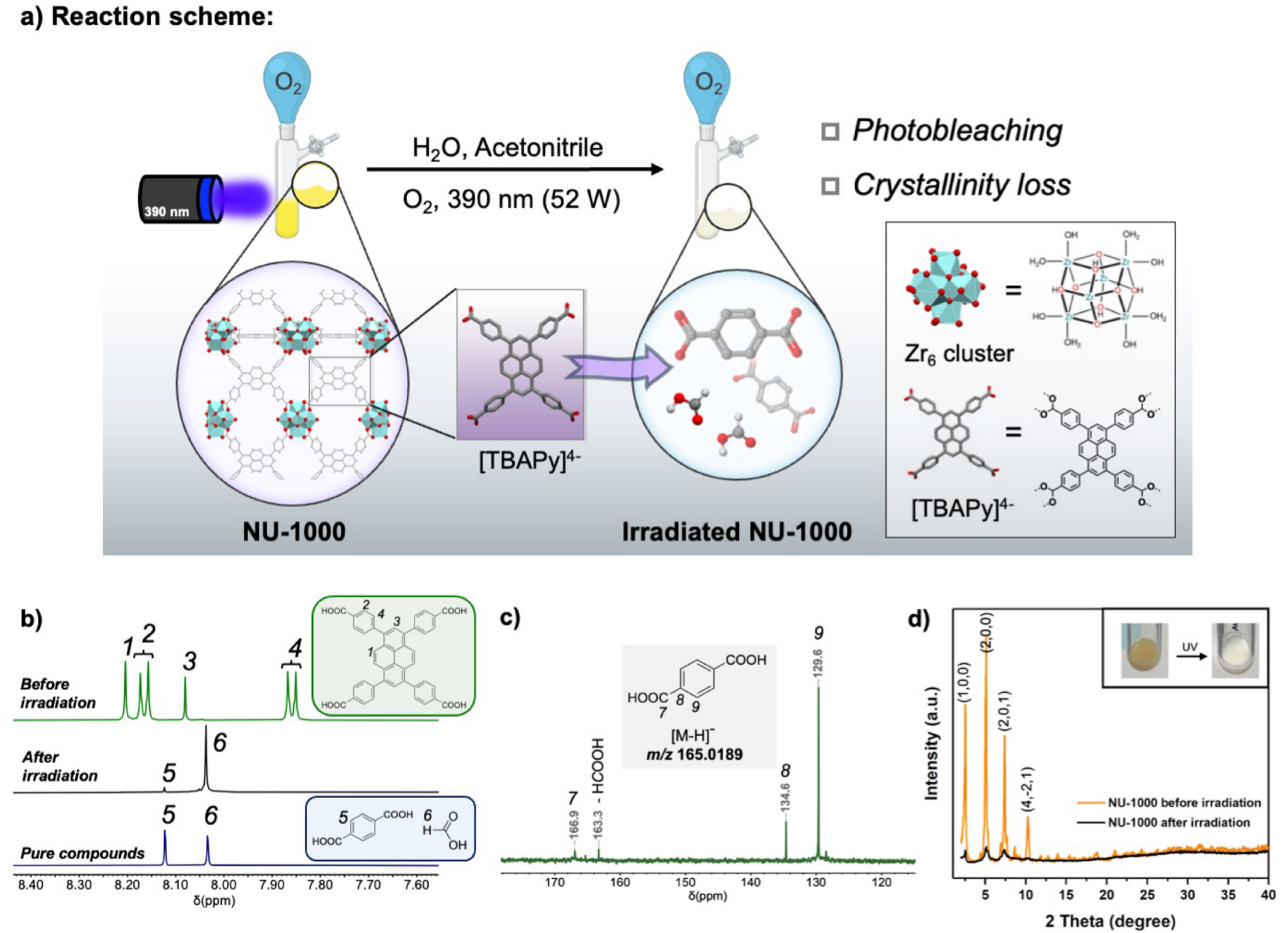
(a) Illustrative scheme of the NU-1000 photodegradation (crystal structure of NU-1000 and ligand are generated from data originally published in ref 1); (b) ^1^H NMR spectra (in *d*_6_-DMSO) of the digested NU-1000 before and after irradiation as well as the ^1^H NMR spectra from pure compounds for comparison; (c) ^13^C­{^1^H}-NMR spectra (in *d*_6_-DMSO) of the NU-1000 after photodegradation, with (inset) the detected mass of the terephthalic acid product by high resolution ESI-MS spectra (negative mode); (d) XRD pattern of NU-1000 sample in acetonitrile exhibits a poor crystallinity after photodegradation and the inset photography shows the color change of the MOF sample after UV irradiation.

To ensure the reproducibility of our results and rule out reactivity arising from trace metal contamination, experiments were conducted independently in two laboratories, using chemicals and glassware from different suppliers. In experiments conducted in the second lab, the formation of H_2_BDC as the final degradation product of NU-1000 under 427 nm LED irradiation with O_2_ was also detected, further confirming the validity and reproducibility of the experimental results (see Figures S16 and S17). Next, we analyzed the residue of the irradiated NU-1000 in MeCN/water mixture by XRD, which showed a significant loss of crystallinity (Figure 3d). Only a trace of characteristic NU-1000 diffraction peaks could be discerned, suggesting that most of the crystalline structure in NU-1000 had collapsed after photodegradation. However, the SEM images in Figure S7 revealed that particle shape and size persisted during the degradation process, as the rod-shape of the MOF was still retained. Considering the highly interconnected coordination framework of the NU-1000, the degraded linkers could still retain the coordination bonds between the Zr nodes and the carboxylate groups, resulting in an amorphous coordination polymer that kept the original external shape of the particles and prevented framework collapse. A similar phenomenon was also reported in the photodegradation of PCN-224-M.^44^ However, when NU-1000 particles were irradiated in DMSO, a solvent known to dissolve the H_2_BDC product, the MOF particles exhibited a slight decrease in average size during the first 7 h of irradiation (Figure S8). After 9 h, some particle aggregation was observed, although the overall shape of the individual particles was still retained. However, as decomposition progressed, the particles ultimately dissolved in DMSO after 18 h.

These subtle surface morphology changes prompted us to further investigate the internal structural transformation of the crystals. N_2_ adsorption measurements at 77 K revealed a decrease in N_2_ uptake for NU-1000 irradiated for 5 h, compared to pristine NU-1000 samples (Figure S6a). We attribute this decrease to pore collapse resulting from TBAPy decomposition. Further irradiation of NU-1000 resulted in an additional decline in N_2_ uptake, indicating further structural degradation (Figure S6b).

We then investigated the possibility of using the H_2_BDC formed from photodegradation of NU-1000 to synthesize UiO-66­(Zr) directly from the crude reaction mixture of irradiated NU-1000 in DMF. All attempts were unsuccessful, evidenced from the absence of the characteristic peaks of UiO-66 in the XRD pattern. This may be due to the low concentration of the H_2_BDC linker compared to conventional synthesis protocols for UiO-66. Nevertheless, SEM images in Figure S9 revealed a different particle morphology, likely resulting from the generation of an unknown phase during the reforming process.

### Conditions for NU-1000 Photodegradation

To identify the critical factors that trigger the photolytic degradation of NU-1000, several experimental conditions were screened, and the results are summarized in Table 1. Since the main observable decomposition product from NU-1000 was identified as H_2_BDC, the relative conversion in Table 1 was calculated by the integral ratio of *n*(H_2_BDC) to *n*(TBAPy) from the ^1^H NMR spectra (Figures S19–S35). Under the standard reaction condition (**Entry 1**) with O_2_ as the mild oxidant in wet MeCN (water content was determined as 1056 ppm by Karl Fischer titration), conversion to H_2_BDC was complete within 18 h of 390 nm irradiation. However, 24% H_2_BDC conversion was observed using dry MeCN (50 ppm water content) (**Entry 2**). No degradation product was detected in the dark (**Entry 3**) or under an inert atmosphere (**Entries 4 and 8**), while replacing the pure O_2_ atmosphere with air (21% O_2_) elicited a lower conversion of 81% (**Entry 5**). Irradiation with light of longer wavelengths greatly reduced the efficacy of photodegradation, with 525 nm light resulting in no discernible H_2_BDC formation (**Entries 11 and 12**). These findings show that NU-1000, as a ligand-based photosensitizer, can only harness light with energy equal to or greater than its HOMO–LUMO gap.^45^ Thus, the combination of water, O_2_ and light of appropriate wavelengths are needed for the complete decomposition of NU-1000.

**Table 1:** Reaction Conditions Screening for the Photodegradation of NU-1000

$\mathrm{NU‐1000}\overset{H_{2}O\left(2.5\mu L\right),\mathrm{Acetonitrile}\left(\mathrm{5 ml}\right)}{\underset{O_{2},30-33\mathrm{{}^{\circ}C},\mathrm{390 nm}\left(\mathrm{52 W}\right),\mathrm{18 h}}{\to }}H_{2}\mathrm{BDC}$		
Entry	Deviation from the standard conditions^a^	Conversion^b^
1	None	100
2	Anhydrous MeCN	24
3	Dark	0
4	Ar atmosphere instead of O_2_	0
5	Air instead of O_2_	81
6	Acetic acid (20 μL, 5 equiv) instead of H_2_O	54
7	H_2_O as solvent instead of MeCN	100
8	H_2_O as solvent instead of MeCN, Ar atmosphere	0
9	DMSO as solvent instead of MeCN	100
10	Anhydrous DMSO	7
11	440 nm instead of 390 nm	30
12	525 nm instead of 390 nm	0
13^c^	H_4_TBAPy (0.01 mol/L) instead of NU-1000, MeCN	0
14^d^	H_4_TBAPy (0.01 mol/L) instead of NU-1000, DMSO	2
15	H_4_TBAPy (0.003 mol/L) instead of NU-1000, DMSO	100
16	H_4_TBAPy (0.003 mol/L) instead of NU-1000, DMSO, 440 nm	16
17	H_4_TBAPy (0.003 mol/L) instead of NU-1000, ZrOCl_2_·8H_2_O (0.4 mg/mL), DMSO	20

a Standard reaction conditions: NU-1000 (20 mg), H_2_O (2.5 μL), solvent (MeCN, 5 mL), 33 °C, 390 nm (52 W), O_2_ balloon, 18 h.

b Conversion was calculated by the relative peak integral ratio of H_2_BDC (singlet peak at 8.04 ppm) to the remaining amount of TBAPy (doublet at 7.78 ppm) in *d*_6_-DMSO by ^1^H NMR using *n*(H_2_BDC)/[*n*(H_2_BDC)+*n*(TBAPy)] (in %).

c H_4_TBAPy powder was used as substrate instead of NU-1000, sparingly soluble in MeCN.

d H_4_TBAPy was used as substrate instead of NU-1000, dissolved in DMSO.

The necessity of H_2_O for the complete decomposition of TBAPy suggests that the degradation of NU-1000 could be reduced, allowing it to be potentially used for photochemical processes for extended periods under anhydrous conditions. However, it is important to note that the nodes of activated Zr-MOFs normally contain coordinated water molecules, which can be replaced by other ligands such as carboxylates,^46^ sulfur oxyanions,^47,48^ or phosphorus oxyanions.^49^ As a result, NU-1000 may release water in the presence of these coordinative ligands. For instance, the addition of acetic acid in place of water in MeCN resulted in 54% degradation of the MOF (**Entry 6**). The amount of acetate group was estimated as 1.5 eq. per Zr_6_ nodes by NMR (see Figure S37). Also, the water content in the reaction mixture was increased from 711 to 766 ppm, implying an exchange between acetic acid in solution and the coordinated water molecules in MOF (Table S1). Therefore, caution is advised when using NU-1000 for oxidation reactions that yield carboxylic acids or oxyacids, as these species may facilitate the release of water, potentially accelerating the photodegradation of the catalyst.

The effect of solvent was further examined by replacing wet MeCN with wet DMSO or water. Like the reaction in MeCN, NU-1000 showed full decomposition with the formation of H_2_BDC, as detected by **^1^** H NMR for both solvents (**Entries 7 and 9**). Notably, irradiation of NU-1000 in wet DMSO resulted in full dissolution of all solids and yielded a clear solution after 18 h (see Figure S18), which is in line with the different solubilities of H_2_BDC in DMSO, MeCN and water. Without external water, the degradation extent in dry DMSO can be suppressed to 7% (**Entry 10**). By choosing DMSO-*d*_6_ as a solvent for the photodegradation allowed for direct analysis of the NMR spectrum without any subsequent workup, again showing H_2_BDC and formic acid as the only NMR-detectable products.

To better understand the role of the porous framework structure of NU-1000 in the photodegradation of the pyrene core of TBAPy, we investigated if other framework structures containing the same pyrene core could also undergo photodegradation. Specifically, we examined whether photodegradation was unique to NU-1000 by studying its structural isomer, NU-901.^41,50^ In addition we explored the reactivity of solid H_4_TBAPy, which was found to crystallize in an open hydrogen-bonded framework, adopting the HOF-101 (also known as PFC-1) structure (see Supporting Information for characterization),^51,52^ as well as H_4_TBAPy in solution.

### Photodegradation of NU-901

Due to differences in the distances between TBAPy linkers, NU-1000 and NU-901 exhibit distinct electronic properties (Figure 4a,b).^53^ To determine whether the observed photodegradation was unique to the NU-1000 polymorph due to its specific electronic structure, we investigated the photodegradation behavior of NU-901.

**Figure 4:**
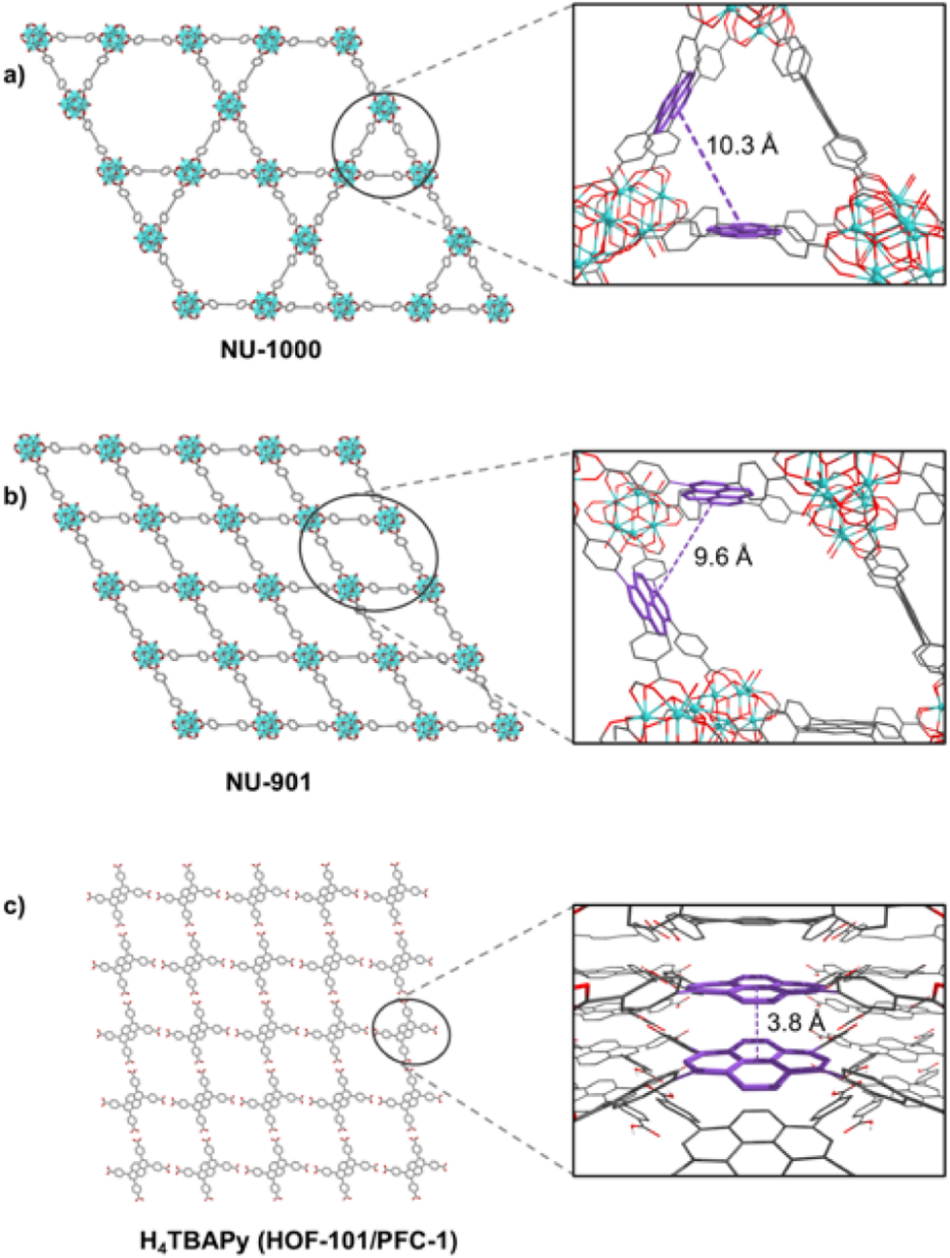
Crystal structures and centroid-centroid distances of pyrene measured from crystal structures of (a) NU-1000,^39^ (b) NU-901 ^50^ and (c) HOF-101/PFC-1.^51^

Phase-pure NU-901 was synthesized and activated following literature procedures.^41^ The purity was confirmed by XRD (Figure S4) and N_2_ adsorption experiments (Figure S6a). Upon irradiation with 390 nm light, under same conditions as Entry 1 in Table 1, NU-901 was found to undergo photodegradation similar to NU-1000, also yielding terephthalic acid as the final product (Figure S2). Time resolved ^1^H NMR analysis of the digested MOFs revealed that the degradation rate of NU-901 was comparable to that of NU-1000 (Figure 5c), hence the photodegradation is not unique to the NU-1000 framework structure.

**Figure 5:**
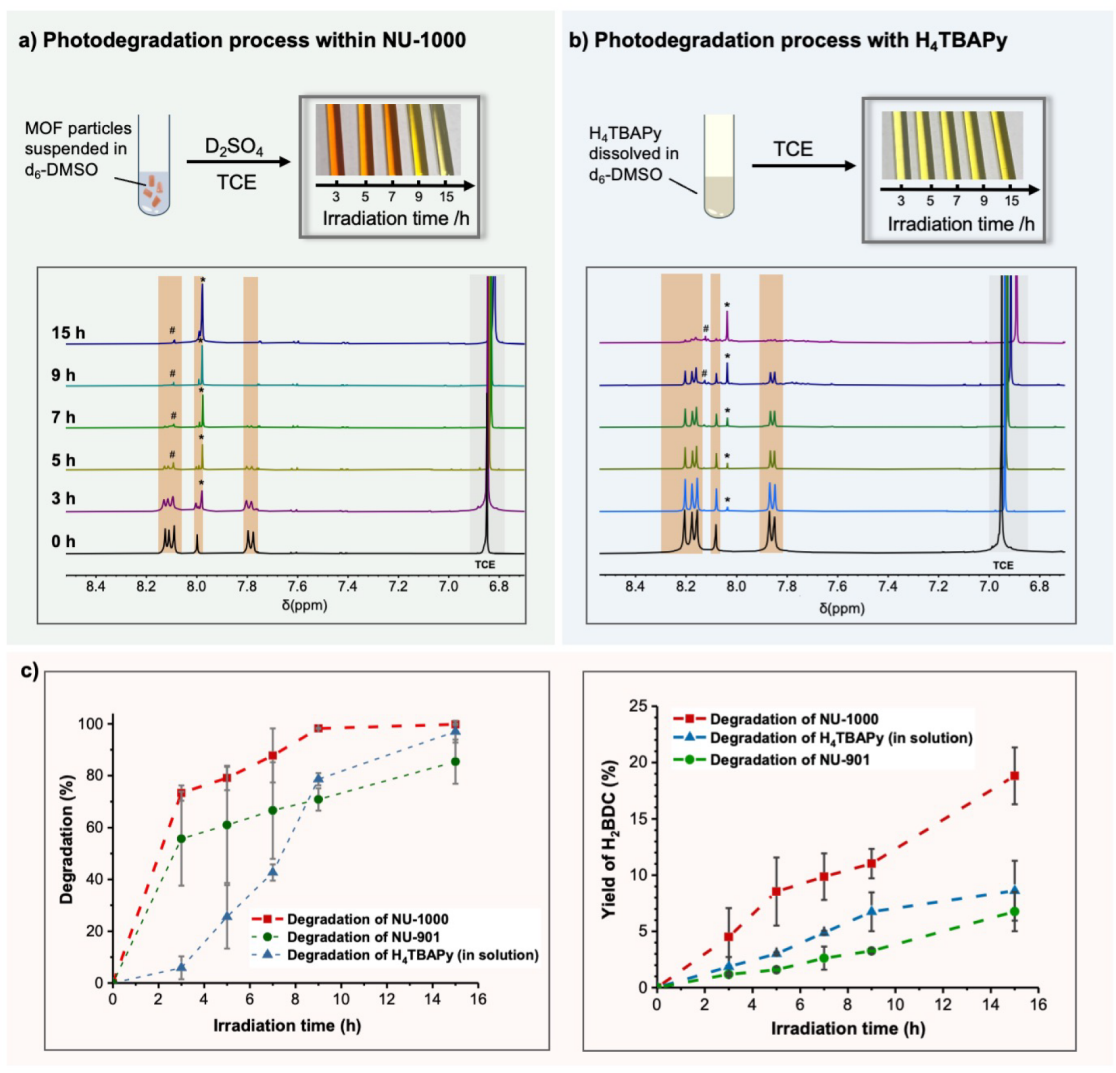
(a) ^1^H NMR monitoring of the progress of the photodegradation in NU-1000 and (b) in its linker molecule H_4_TBAPy (3 mmol/L in DMSO) after a certain irradiation time. The photography shows the color change of the crude product mixture in DMSO during the irradiation. The peaks highlighted in orange were assigned to the TBAPy. Formic acid was indicated with # and terephthalic acid with *, respectively; (c) Comparison of the ligand degradation extent (left) and the H_2_BDC yield (right) in NU-1000 (red squares), in NU-901 (green circles) and in the dilute solution of H_4_TBAPy (3 mmol/L in DMSO) (blue triangles) over the irradiation process.

### Photodegradation of H_4_TBAPy in Solid State and Solution

XRD analysis of the H_4_TBAPy powder used in all experiments confirmed that it crystallized in the HOF-101 structure (Figure S5), which is porous but exhibits π-stacking between the pyrene rings (Figure 4c), a feature absent in NU-1000 and NU-901 (Figure 4a,b). We further studied the photodegradation behavior of HOF-101 to determine whether π-stacking in the pure ligand could suppress degradation despite its open-framework structure. We also examined the degradation of H_4_TBAPy in solution, where π-stacking would be eliminated at low concentrations.

First, H_4_TBAPy (HOF-101) powder, which is insoluble in MeCN, yielded no photodegradation when irradiated as a suspension in MeCN (Table 1 **Entry 13**). When H_4_TBAPy was dissolved in DMSO, concentration-dependent photodegradation efficacy was observed (**Entries 14 and 15**), with low H_4_TBAPy concentration (3 mM) eliciting complete decomposition. Dilution studies of H_4_TBAPy in *d*_6_-DMSO revealed that at lower concentrations, the pyrene ^1^H NMR signals appeared more deshielded (Figure S36), indicating π-π stacking at higher concentrations.^54^ Based on the unit cell volume of NU-1000 and the number of linkers, the concentration of TBAPy within NU-1000 was calculated to be 0.448 mol/L (see Supporting Information), which is significantly higher than the concentrations used in **Entries 13 and 14**. Taken together, these observations underscore the necessity for sufficient spatial separation between pyrene cores, as π-stacking in both HOF-101 and concentrated solution appears to hinder the photoreactivity

In a similar way as in NU-1000, irradiation of a H_4_TBAPy solution in DMSO at a longer wavelength (440 nm) reduced the extent of photodegradation (**Entry 16**). Furthermore, a solution of H_4_TBAPy and ZrOCl_2_·8H_2_O in DMSO afforded only a small extent of conversion to H_2_BDC (**Entry 17**), indicating that the Lewis acidity of Zr was not the determining factor for the NU-1000 degradation. The kinetics of photodegradation of NU-1000 and H_4_TBAPy in *d*_6_-DMSO were compared though time-course quantitative ^1^H NMR experiments using 1,1,2,2-tetrachloroethane (TCE) as an internal standard (see Supporting Information for detailed procedure). The extent of photodegradation was determined according to the following equation:

$$\mathrm{Degradation}\left(\%\right)=\frac{N_{0}-N_{I}}{N_{0}}\times 100\%$$

where *N*_0_ = original molar amount of the ligand, *N*_I_ = molar amount of ligand irradiated at time t. As shown in Figure 5a,b, the signals from the H_4_TBAPy linkers (marked in orange) gradually disappeared, with the appearance of H_2_BDC and formic acid as degradation products. No other products were formed in significant amounts, and thus only the yield of H_2_BDC was quantitated. When immobilized within the NU-1000 framework, more rapid degradation of the linker was evident. After 3 h of 390 nm irradiation, 78% of the pyrene unit in NU-1000 was degraded, compared to only 3% in the homogeneous linker solution. Notably, nearly 100% of the TBAPy ligand was decomposed in both the homogeneous solution and within the MOF scaffold after 15 h. However, the yield of H_2_BDC was significantly lower in the homogeneous solution (6%) compared to the reaction in MOF reaction (17%) (Figure 5). The ^1^H NMR spectrum for the dissolved TBAPy ligand after 15 h revealed unidentifiable aromatic side products, but this was not observed for NU-1000. This suggests that photodegradation in NU-1000 is more efficient with fewer side products generated other than H_2_BDC and formic acid. Considering that the formation of H_2_BDC from TBAPy is likely caused by the complete oxidation of the pyrene core, which is expected to yield four molar equivalents of H_2_BDC from the ligand’s four phenyl carboxylate groups, the low yields in H_2_BDC may be attributed to overoxidation to gaseous products.^35,55^

To determine the mass changes caused by the evolution of volatile products, NU-1000 powder was irradiated in the absence of any solvent under air (Figure S12), and thermogravimetric analysis (TGA) was conducted on both pristine and irradiated NU-1000 (Figure S13). Under these ambient conditions, moisture in the air may gradually diffuse into the MOF, contributing to the degradation process. The results show that the irradiated MOF contains significantly less organic material compared to pristine NU-1000.

### Mechanistic Study of the NU-1000 Photodegradation

We next aimed for a better understanding of the mechanism underlying the photodegradation of NU-1000. Initially, we hypothesized that the reactive oxygen species (ROS) generated by NU-1000 under irradiation would be responsible for attacking the pyrene rings of the MOF, given that ROS, such as singlet oxygen, are highly reactive toward organic molecules.^21–26^ However, experimental data showed that solvents and additives such as DMSO and acetic acid, which typically react with ROS, did not inhibit NU-1000 degradation. Notably, DMSO, which is expected to scavenge both singlet oxygen (^1^O_2_),^56^ and open shell ROS,^57,58^ failed to inhibit degradation, suggesting that ROS are not directly responsible for the degradation of the MOF.

Instead, we hypothesized that the reactive species originates within the MOF itself, possibly in the form of a radical hole that reacts with nucleophiles such as water. This radical hole may be generated via a single electron transfer from NU-1000 to oxygen, producing superoxide (O_2_•^–^) and a radical hole localized on the pyrene core, which can also hop along the channel direction.^59^ To test this hypothesis, we used *N,N,N’,N’*-tetramethyl phenylenediamine (TMPD) as a probe. Photoelectron transfer from excited NU-1000 (NU-1000*) to oxygen generated superoxide and a radical cation of NU-1000 (NU-1000•^+^), which subsequently oxidized TMPD to form Wurster’s blue (TMPD•^+^), identified by its characteristic absorption at 612 nm. Indeed, irradiating NU-1000 in MeCN/H_2_O in the presence of TMPD yielded TMPD•^+^, which was monitored by UV–vis spectroscopy, as shown in Figure 6.

**Figure 6:**
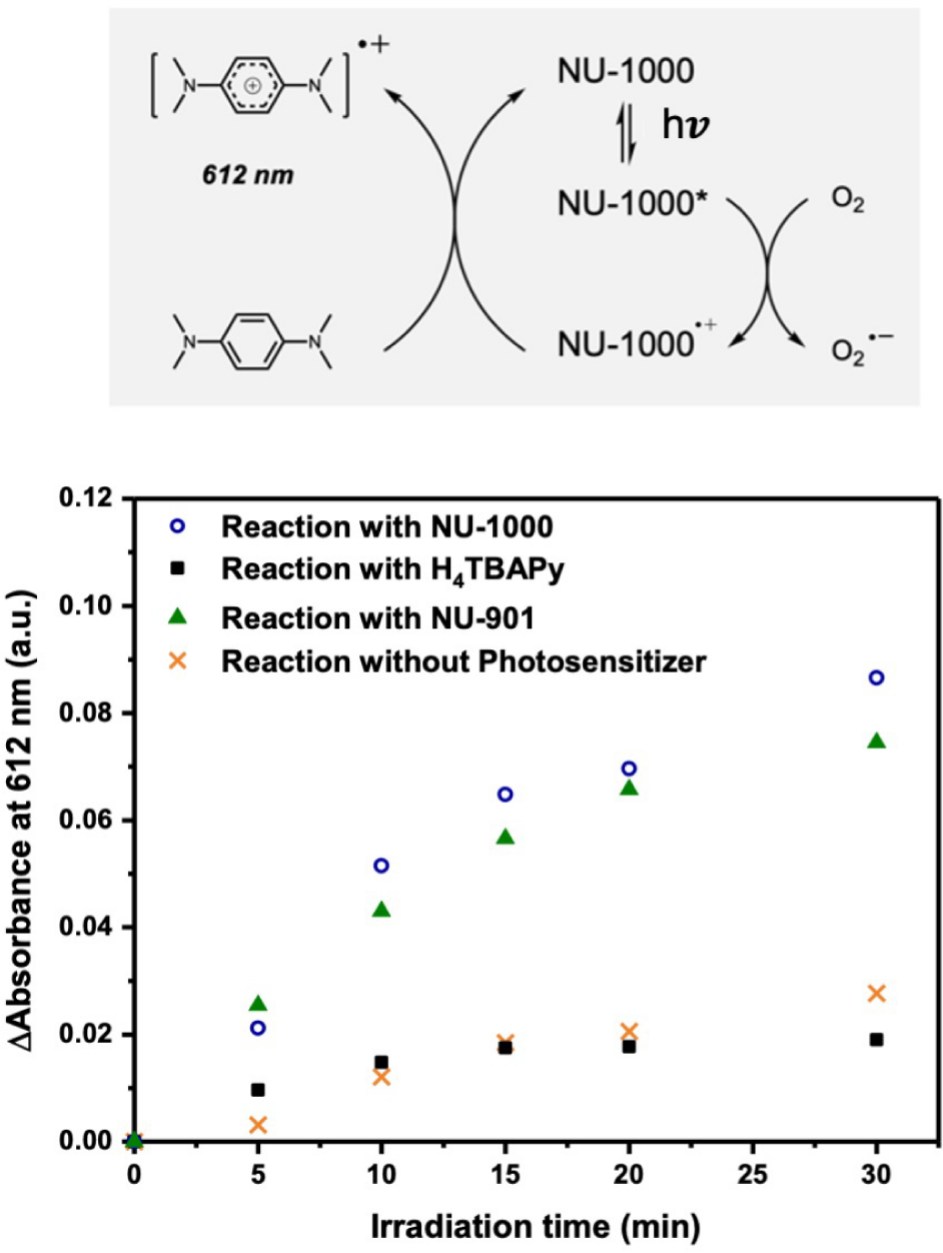
Comparison of the capacity in generating superoxide among NU-1000 (blue circles), H_4_TBAPy (black squares), NU-901 (green triangle) and blank (orange crosses). MOF and H_4_TBAPy were used as photocatalyst, while TMPD as substrate, added to a solvent mixture of MeCN and H_2_O (1:1, *v:v*). The reaction was irradiated at 390 nm in air. The product formation was monitored by the maximal absorbance at 612 nm. ΔAbsorbance was calculated as *A*(*t*) *– A*(*t* = 0).

We also investigated the oxidation of TMPD using NU-901 and pure H_4_TBAPy. Upon irradiation, NU-1000 and NU-901 exhibited a more rapid single electron transfer compared to H_4_TBAPy, correlating with their faster rate of degradation, shown in Figure 5c.

Interestingly, the presence of TMPD was found to inhibit NU-1000 degradation, implying that hole scavengers can effectively protect the MOF from photodegradation. We tested other single-electron donors, including DABCO, TEMPO radical, triethylamine and 1,5-dihydroxylnaphthalene, all of which inhibited NU-1000 degradation significantly (see Table S4 and Figures S38–S40, S45 and S46). Given that tertiary amines were found to inhibit the photodegradation of NU-1000, we finally explored if the use of HEPES (4-(2-hydroxyethyl)-1-piperazineethanesulfonic acid), a commonly used biological buffer agent, could also protect MOF from degradation. HEPES was indeed found to have a protective effect (Figure S47), suggesting a potential benefit of using an amine-containing buffer system with NU-1000 when used for biological applications.

The proposed mechanism involving the formation of superoxide (O_2_^•–^) and a pyrene-centered radical hole aligns with the observation that water is essential for effective degradation. Under anhydrous conditions, O_2_^•–^ is relatively stable, but it reacts rapidly with water via deprotonation to yield hydroxide ions (OH^–^) and hydroperoxyl radicals (HOO^•^).^60–62^ Concurrently, the carbon-centered radical hole on NU-1000 would be expected to react rapidly with the OH^–^ formed, yielding a hydroxylated neutral radical on the pyrene. This product is analogous to the reaction of a hydroxyl radical with ground state pyrene (Scheme 1). We postulated that the reaction then proceeds via successive addition of triplet O_2_, until the terminal product H_2_BDC is formed, similar to ^•^OH radical initiated oxidation of other aromatics.^63,64^ Meanwhile, we expect that the HOO^•^ radical formed reacts with the solvent. However, it is possible that the ROS species formed contribute to the further oxidation of fragments generated after the initial cleavage of the pyrene ring.

**Scheme 1:**
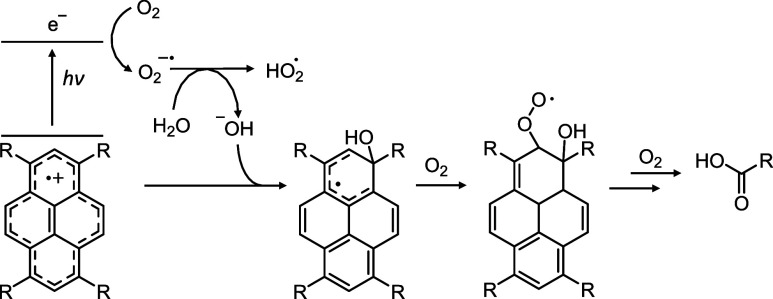
Proposed Route for NU-1000 Photodegradation in the Presence of Water and Oxygen

To further test our proposed mechanism, we examined additional ROS scavengers, specifically methanol and styrene. NU-1000 underwent photodegradation in the presence of both, reinforcing that ROS scavengers alone may not prevent degradation unless they also function as single-electron donors.

When methanol was used as a solvent, NU-1000 underwent extensive photodegradation, producing a complex mixture of aromatic products beyond H_2_BDC (Figure S41). Although methanol is a known radical scavenger,^65^ it is also expected to react rapidly with superoxide,^60–62^ similar to how water functions in our previous reactions. This reaction generates strong nucleophiles capable of attacking the pyrene core. Similarly, styrene, which is expected to react with ^1^O_2_,^66^ provided no protection against photodegradation (Figure S42).

The observation that single-electron donors prevent NU-1000 from degrading provides valuable insight into its potential as a photocatalyst for specific substrates. The suitability of NU-1000 as a photocatalyst would depend on the oxidation mechanism involved. If the reaction proceeds via hole transfer to the substrate, the MOF structure remains intact. In this case, substrates capable of undergoing single-electron oxidation by the photoexcited NU-1000 hole could be efficiently processed without compromising the stability of the framework. A notable example of such a substrate are derivatives containing thioether functional groups (e.g., mustard gases), which are known to undergo one-electron oxidation.^67,68^

Furthermore, the production of singlet oxygen (^1^O_2_) should not lead to MOF degradation, as this pathway does not generate holes within the MOF itself. However, intersystem crossing in NU-1000 is known to be slow,^69^ potentially limiting the efficiency of this mechanism.

## Conclusion

In summary, our study explored the photodegradation pathway of NU-1000 upon photoirradiation. A series of control experiments demonstrated that the complete decomposition of the linker molecule TBAPy requires oxygen, H_2_O and light of appropriate energy. Also, molecules capable of coordination, such as carboxylic acid that can compete with the coordinated water in MOF may also potentially cause the release of free water molecule that can favor NU-1000 degradation. Time-resolved NMR studies showed a full dissolution of NU-1000 in DMSO after 15 h of irradiation with terephthalic acid identified as the main degradation product. The decomposition of TBAPy occurred more efficiently in NU-1000 than in its homogeneous solution, due to the rapid formation of highly reactive species. Moreover, the highly porous structure in MOF provides rapid diffusion pathways, allowing substrates and reactive oxygen species to interact more efficiently.

Considering the widespread use of NU-1000 in various applications, like gas adsorption, separation, sensing and a wide range of photocatalytic reactions, our findings highlight the importance in understanding the photochemical decomposition of these pyrene-based materials. In particular, the involvement of ubiquitous reagents such as water, oxidants and coordinating compounds in NU-1000 photodegradation necessitates taking them into account for any real-life practical applications. Additionally, the photoinduced degradation of NU-1000 under mild conditions presents a potential environmental benefit, helping to mitigate long-term accumulation in natural settings. This dual insight into stability and environmentally responsive degradation pathway of NU-1000 serves as valuable guidance for both enhancing its useful lifetime and sustainable design in MOF applications.

## Supplementary Material



## Notes

Additional experimental section, supplementary figures and tables, and supplementary discussion (PDF)
