# Supplementary material for: Aerobic Photodegradation of Pyrene-Based Metal–Organic Framework NU-1000 to Terephthalic Acid
Source: Chem Mater. 2025 May 2;37(9):3531–41. doi: 10.1021/acs.chemmater.5c00520 (PMC13155651; doi:10.1021/acs.chemmater.5c00520)
Supplement: Supplementary file 1 [file cm5c00520_si_001.pdf]

Supporting Information

Aerobic Photodegradation of Pyrene-based Metal-Organic Framework NU-1000 to Terephthalic Acid

Wenyi Zeng,[a, b] Tristan T. Y. Tan,*[a] Youven Benseghir,[c] Michael R. Reithofer,[b] Jia Min Chin*[c] and Jason Y. C. Lim*[a,d]

[a] Institute of Materials Research and Engineering (IMRE), Agency for Science, Technology and Research (A*STAR), 2 Fusionopolis Way, Innovis #08-03, Singapore 138634, Republic of Singapore

[b] Institute of Inorganic Chemistry, Faculty of Chemistry, University of Vienna, Währinger Str. 42, 1090 Vienna, Austria

[c] Institute of Functional Materials and Catalysis, Faculty of Chemistry, University of Vienna, Währinger Str. 42, 1090 Vienna, Austria

[d] Department of Materials Science and Engineering, National University of Singapore (NUS), 9 Engineering Drive, Singapore 117576, Republic of Singapore

Table of Contents

Materials and Methods 2

Additional Experimental Details 4

Time-resolved NMR studies 4

Power dependency study 5

Characterization Data 6

X-ray diffraction data 6

Nitrogen adsorption isotherms of NU-1000 and NU-901 8

SEM Images 9

FT-IR Spectra 11

Diffuse Reflectance UV-Vis Spectra 12

Additional Photodegradation Experiments 13

Characterization of the NU-1000 photodegradation product 16

Characterization data from photodegradation experiment conducted at the University of Vienna 18

NMR Spectra of Photodegradation Experiments 19

Reference 35

Materials and Methods

$ZrOCl_2 \cdot 8H_2O$ (98%, Merck), trifluoroacetic acid (TFA, Sigma-Aldrich), triethylamine (Alfa Aesar), benzoic acid (Sigma-Aldrich), 1,4-diazabicyclo[2,2,2]octane (DABCO, Alfa Aesar), 2,2,6,6-tetramethylpiperidine-1-oxyl (TEMPO, Sigma-Aldrich), *N*,*N*,*N*',*N*'-tetramethyl phenylenediamine (TMPD, Sigma-Aldrich), 1,5-dihydroxynaphthalene (Sigma-Aldrich), Zirconium acetylacetonate (Sigma-Aldrich), 4-aminobenzoic acid (Sigma-Aldrich), 4-(2-Hydroxyethyl)piperazine-1-ethanesulfonic acid (HEPES, Sigma-Aldrich), N,N-dimethylformamide (Sigma-Aldrich), acetonitrile (gradient grade, Sigma-Aldrich), dimethyl sulfoxide (DMSO, Kanto Chemical), anhydrous acetonitrile (99%, Sigma-Aldrich) and anhydrous DMSO (99%, Sigma-Aldrich) were purchased and used without further purification. 4,4',4'',4'''-(pyrene-1,3,6,8-tetrayl) tetrabenzoic acid (H_4TBAPy) was synthesized according to the literature.[1] Dimethyl sulfoxide-D_6 (D, 99.9%) and deuterium oxide (D, 99.9%) were purchased from Cambridge Isotope Laboratories. Light emitting diodes (LEDs - 390 nm, 427 nm, 440 nm and 525 nm) were purchased from Kessil, PR160L.

X-ray diffraction (XRD) data were collected using a Bruker D8-Advance X-ray diffractometer with Cu Kα (λ = 1.5406 Å) radiation, at a resolution of 0.02° and a scan rate of 0.1 s/point.

Scanning electron microscopy (SEM) images of the MOF particles were captured on a JEOL JSM6700F with 5.0 kV accelerating voltage and 10 µA emission current. The sample powder was loaded on a carbon tape and sputter coated with gold before measurement.

Nuclear magnetic resonance (NMR) was recorded with a JEOL 500 MHz spectrometer (Tokyo, Japan) in d_6-DMSO or in D_2O. The chemical shift was measured in ppm. Repeat experiments in Vienna (Figure S10-11) were recorded on Bruker BioSpin AV neo 500 (^{1}H-NMR: 500.32 Hz, ^{13}C-NMR: 125.81 Hz) provided by the NMR Center, Faculty of Chemistry, University of Vienna.

Electrospray Ionization Mass spectrometry (ESI-MS) was conducted on a Bruker timsTOF flex spectrometer at Mass Spectrometry Centre, Faculty of Chemistry, University of Vienna. The high-resolution mass spectrum was recorded by using MALDI with DCTB as the matrix for laser desorption and ionization.

Solution state UV-Vis spectroscopy was performed with a SHIMADZU UV-Vis recording spectrophotometer (UV-2501PC). Sample solution was measured in a polymethyl methacrylate (PMMA)-based disposable cuvette and data were recorded with the software UVProbe 2.70.

Thermogravimetric analysis (TGA) was performed to observe the mass loss during the thermal decomposition of MOF samples under air. The experiments were carried out in an Q500 V6.7 Build 203 analyzer (TA Instruments) at a heating rate of 5 °C/min, from room temperature to 800 °C with a flow rate of 40 mL/min. In each experiment, about 5 mg sample was placed in alumina crucibles.

Fourier-transform infrared spectroscopy (FTIR) was measured with VERTEX 80/80v FT-IR spectrometer (Bruker) with attenuated total reflection (ATR). The scan range was 4000-400 cm^{-1}. Dry powder sample was placed in contact with a high-refractive-index crystal

Nitrogen adsorption-desorption isotherm was recorded by using a Micromeritics ASAP 2460 Analyzer. Prior to the measurements, the samples were degassed at 0.01 mbar, 150 °C for 10 h.

Solid state UV-Vis spectra were recorded using a Shimadzu UV-vis spectrometer 3600 with integrating sphere attachment.

Karl Fischer (KF) Titration was used to determine the water content in acetonitrile and dimethyl sulfoxide. The measurement was conducted with C20 Compact Karl Fischer Coulometer and calibrated with HYDRANAL™ Water Standard 0.1 PC.

Table S1: Determination of water content in different solvent systems by Karl Fischer Titration.

Sample	Water content (ppm)
MeCN (5 mL) + H_2O (2.5 µL)	1056.0
Anhydrous MeCN	50.0
Acetic acid (20 µL) + MeCN (5 mL)	223.1*
[Anhydrous MeCN + NU-1000 + Acetic acid], stirred overnight	766.4**
[Anhydrous MeCN + NU-1000] + Acetic acid	711.3***

*Non-anhydrous acetic acid was used.

,* To explore the substitution of coordinated H_2O in NU-1000 by acetic acid, the water content from two samples were compared. (*) NU-1000 (4 mg), acetic acid (4 µL), anhydrous MeCN (1 mL) were stirred at room temperature overnight. Then the supernatant was submitted to KF Titration Test. (**) NU-1000 (4 mg) was stirred in anhydrous MeCN (1 mL) at room temperature overnight. Then acetic acid (4 µL) was mixed with the supernatant before submitted to KF Titration Test.

LED power density

The average intensity of the Kessil PR160L series lamp at 6 cm distance is 137 mW/cm^2, as detailed in the manufacturer's specifications. Since the intensity of light (I) is inversely proportional to r^2 (r = distance). The power density in this experiment can be estimated as

$$r_{exp} = 1.67 \times r_{@6cm}$$

$$I_{exp} = \frac{1}{(1.67)^2} \times I_{@6cm}$$

$$I_{exp} = 49 \text{ mW/cm}^2$$

Temperature stability of reactions

The temperature of the reactions was controlled as previous report.[2] Briefly, the reaction was maintained to near ambient temperature using an air stream supplied by a small fan, and the reaction temperature was recorded at various time intervals shown in **Figure S1**.

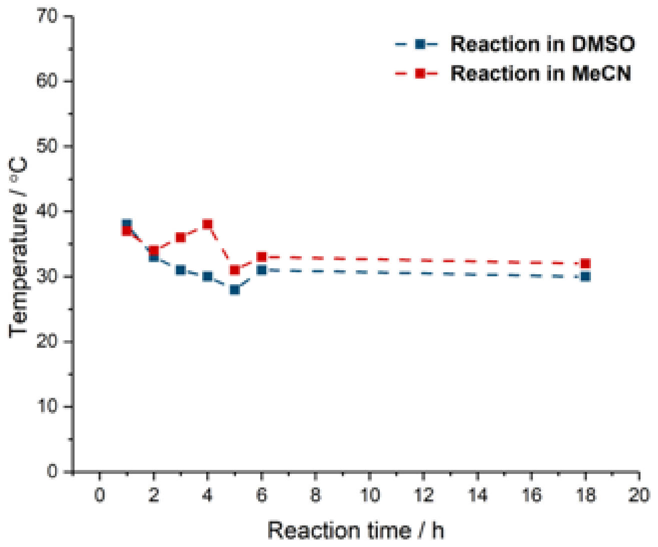

Figure S1: Temperature change of the reaction mixture (Acetonitrile and DMSO) during the photoirradiation.

Additional Experimental Details

Attempted Synthesis of UiO-66(Zr) from the crude reaction mixture of NU-1000 photodegradation

NU-1000 (20 mg, 9.19 µmol) was first irradiated with 390 nm LED in DMF (5 mL) with DI H_2O (2.5 µL), at 30 °C, under O_2 atmosphere for 18 h. Then the reaction crude was further heated at 120 °C in oven for overnight in an attempt to synthesize UiO-66(Zr). After the reaction, the solid part was separated from the reaction mixture via centrifugation, washed twice with acetone and dried in vacuum for SEM measurement.

Time-resolved NMR studies

Reaction with NU-1000: In a 20 mL vial, activated NU-1000 (20 mg, 9.19 µmol) was suspended in a mixture of d_6-DMSO (5 mL) and DI water (2.5 µL). The reaction mixture was irradiated and stirred under 390 nm at 30 °C, O_2. At specified reaction time points, 500 µL aliquot from the reaction suspension was collected, added with D_2SO_4 (25 µL) and TCE (1 µL, as internal standard), forming a clear solution for ^{1}H-NMR measurement.

Reaction with H_4TBAPy: In a 20 mL vial, H_4TBAPy (10 mg, 14.65 µmol) was dissolved in a mixture of d_6-DMSO (5 mL) and DI water (2.5 µL). The reaction mixture was irradiated and stirred under 390 nm at 30 °C, O_2. At specified reaction time points, 500 µL aliquot from the reaction suspension was collected and TCE (1 µL) was added as internal standard for ^{1}H-NMR measurement.

Reaction with NU-901: In a 20 mL vial, NU-901 (20 mg, 9.19 µmol) was suspended in a mixture of d_6-DMSO (5 mL) and DI water (2.5 µL). The reaction mixture was irradiated and stirred under 390 nm at 30 °C, O_2. At specified reaction time points, 500 µL aliquot from the reaction suspension was collected, added with D_2SO_4 (25 µL) and TCE (1 µL, as internal standard), forming a clear solution for ^{1}H-NMR measurement.

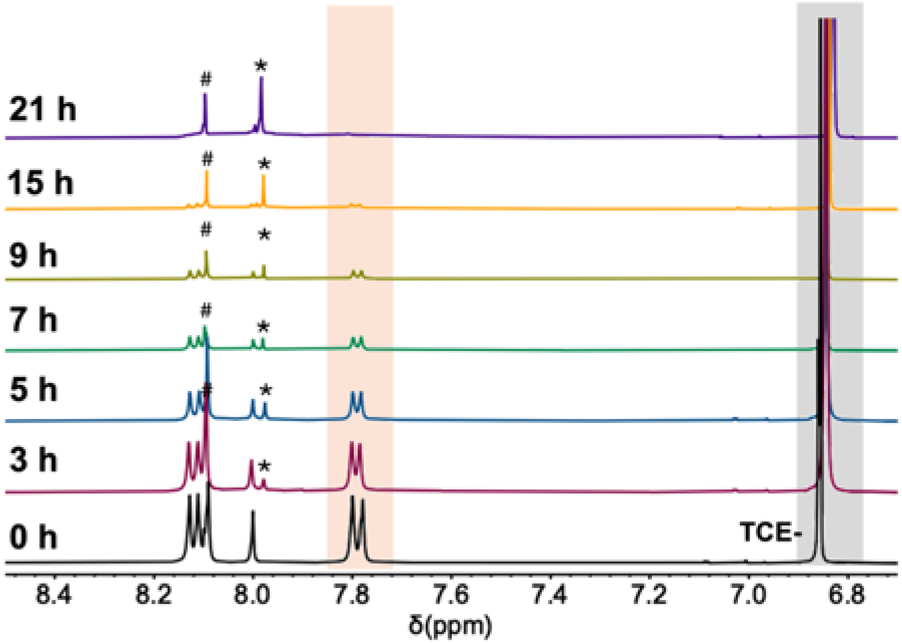

Figure S2: ^{1}H-NMR monitoring of the progress of the photodegradation in NU-901 in DMSO after a certain irradiation time. The peaks highlighted in orange was assigned to the TBAPy. Formic acid was indicated with # and terephthalic acid with *, respectively.

Power dependency study

The dependence of NU-1000's photodegradation rate on LED power was investigated under the same reaction conditions as **Entry 1** in **Table 1**, with the power output adjusted using the LED power control. As shown in **Table S2**, photodegradation decreased as the LED power was reduced.

Table S2: NU-1000 photodegradation dependency on LED power

LED Power	Relative degradation [%]
25% (13 W)	6
50% (26 W)	11
100% (52 W)	100

Characterization Data

X-ray diffraction data

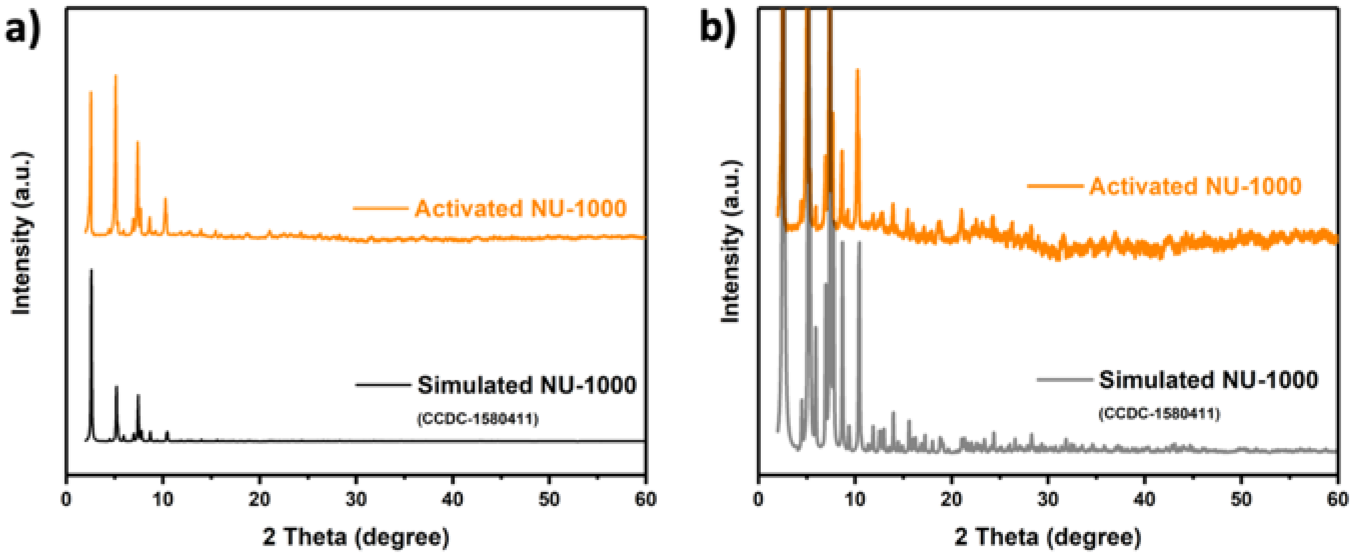

Figure S3: XRD patterns of the activated NU-1000 (a) and the zoom view of the y-axis region (b).

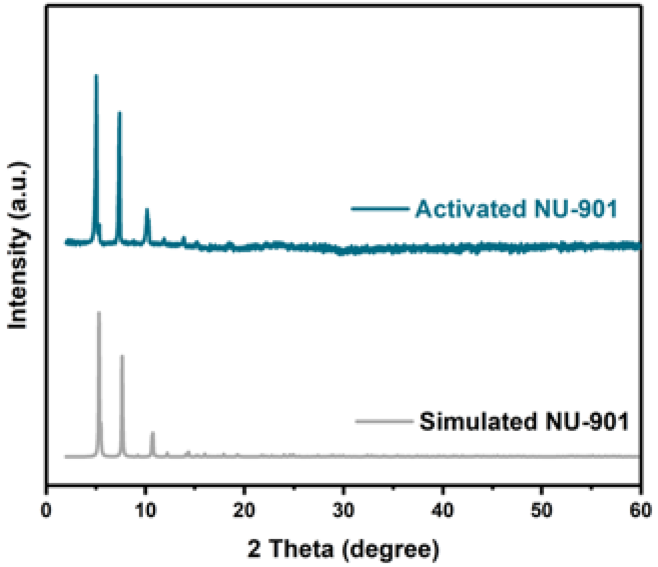

Figure S4: XRD patterns of the activated NU-901 and the simulated NU-901[3].

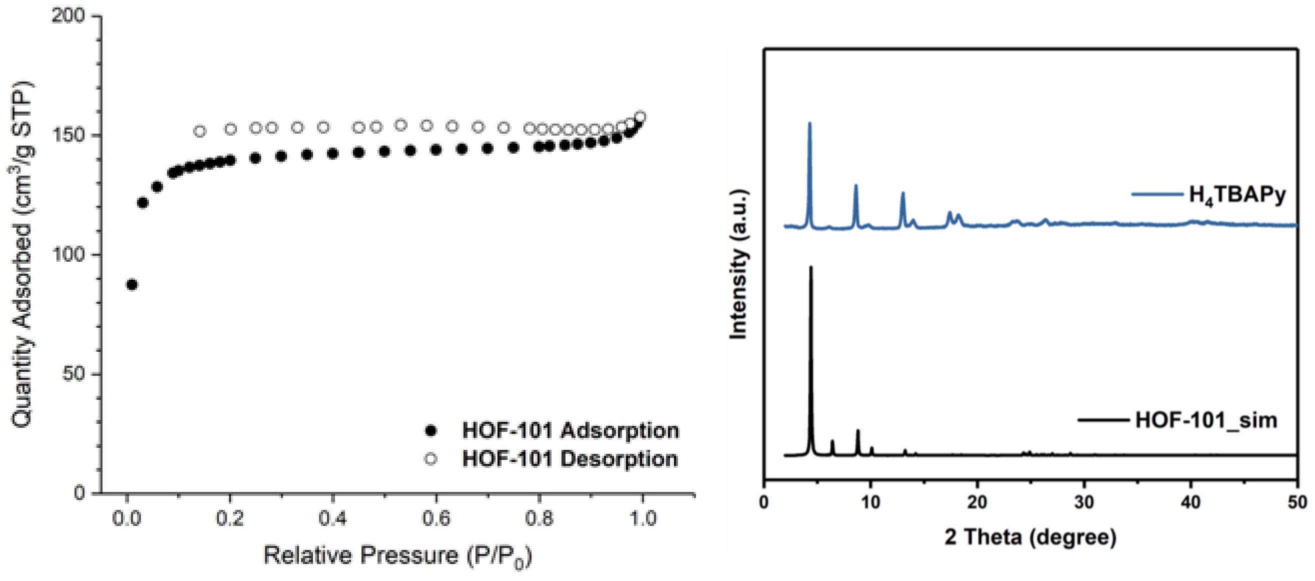

Figure S5: Nitrogen adsorption-desorption isotherms (left) and the XRD pattern (right) of H_4TBAPy.[4]

Nitrogen adsorption isotherms of NU-1000 and NU-901

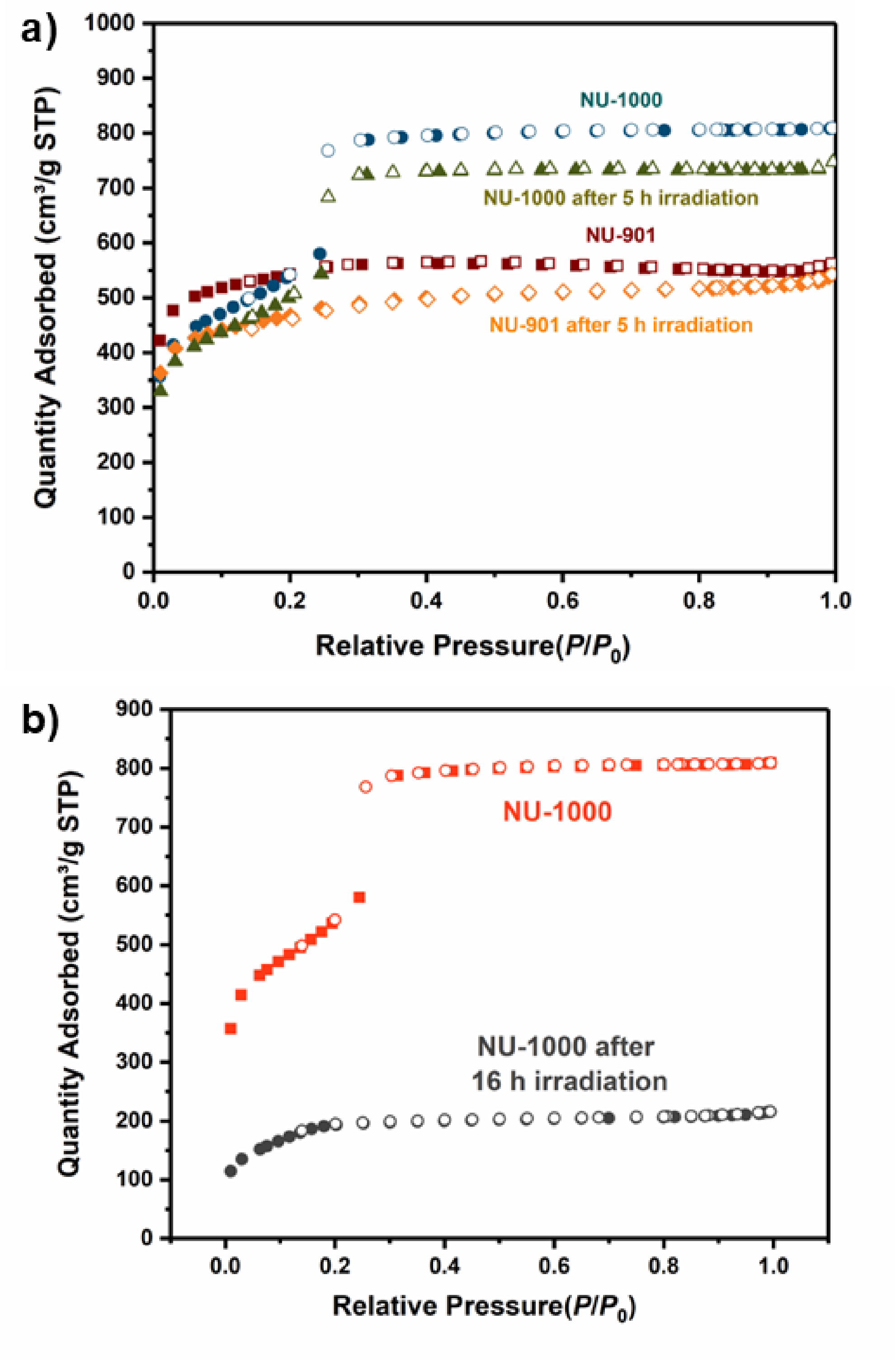

Figure S6: a) Nitrogen adsorption-desorption isotherms of pristine NU-1000 (in blue), NU-1000 after 5 hours (in green), NU-901 (in red) and NU-901 after 5 hours (in orange) (reaction condition same as the entry 8 in Table 1), b) Comparison of N_2 isotherm of pristine NU-1000 and NU-1000 after 16 hours of irradiation.

SEM Images

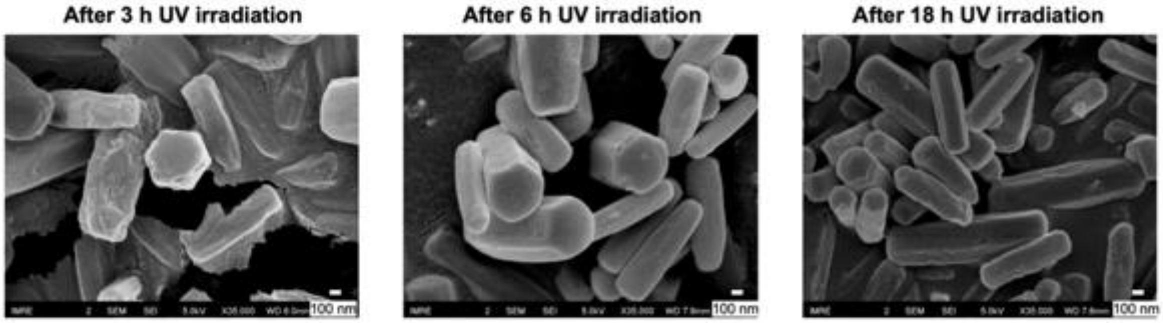

Figure S7: SEM images of NU-1000 after irradiation with 390 nm in acetonitrile.

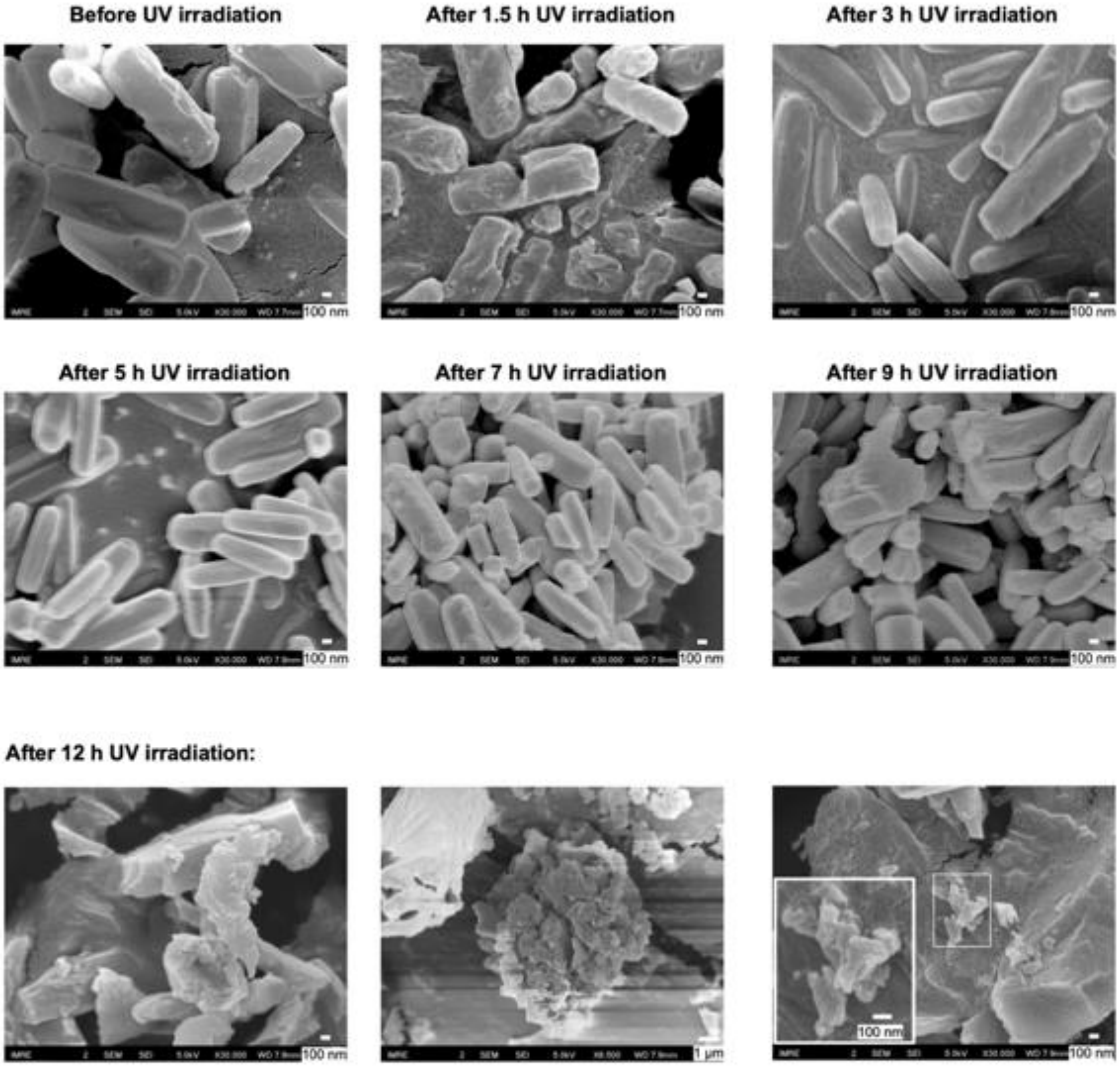

Figure S8: SEM images of NU-1000 before and after irradiation with 390 nm in DMSO.

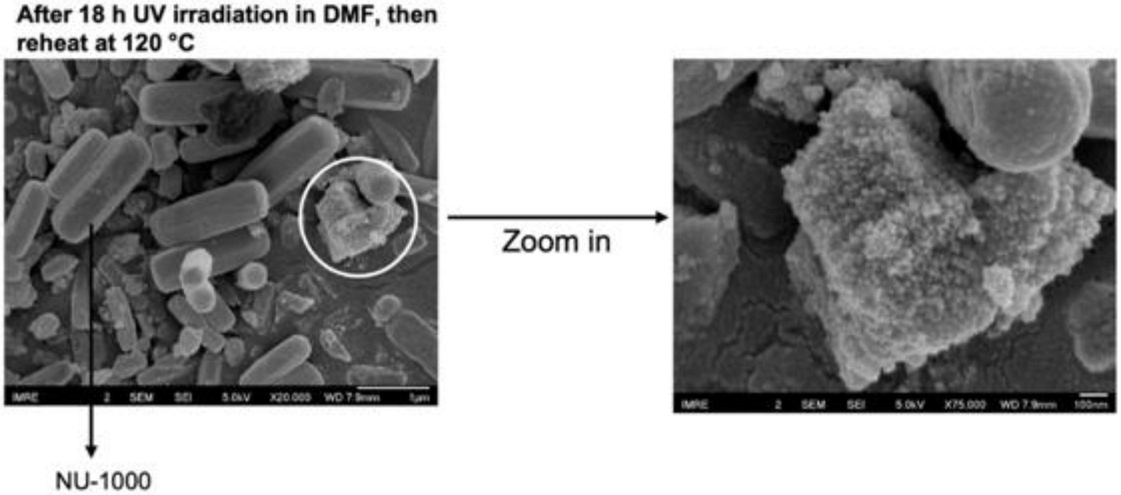

Figure S9: SEM image of NU-1000 irradiated and further heated in DMF. NU-1000 (20 mg, 9.19 µmol) was first irradiated in DMF (5 mL) with DI H_2O (2.5 µL), 30 °C, 390 nm, O_2, 18 h, then the reaction crude was further heated at 120 °C for overnight in an attempt to synthesize UiO-66(Zr). After the reaction the solid was separated from the reaction mixture, washed with acetone and dried in vacuum before taking SEM measurement.

FT-IR Spectra

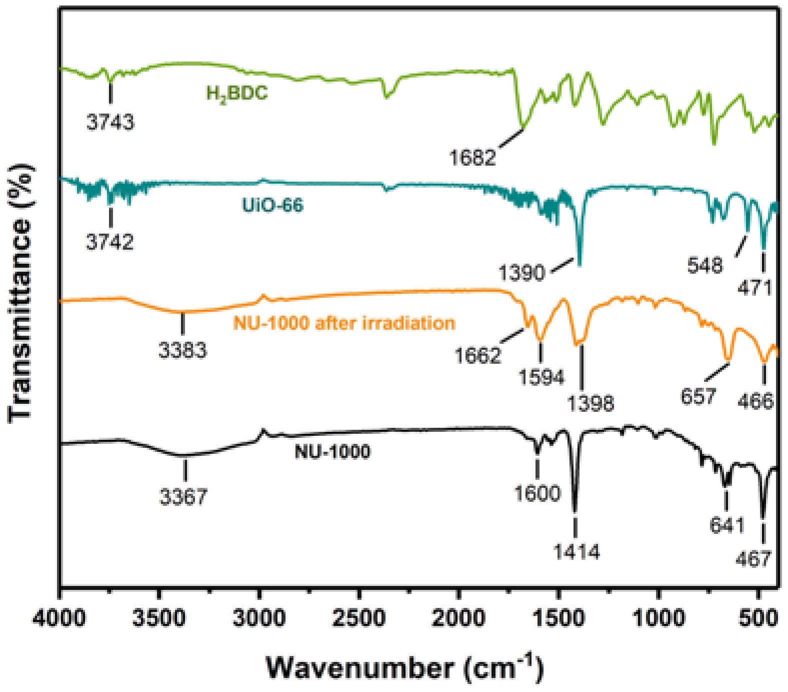

Figure S10: FT-IR spectrum of NU-1000 before and after irradiation, terephthalic acid, and UiO-66.

Table S3: FT-IR assignment of NU-1000, NU-1000 after irradiation, UiO-66 and H_2BDC in Figure S10.

Wavenumber (cm^{-1})	Assignment
3743, 3742, 3383, 3367	O-H stretching
1682,1662	C=O
1594, 1600	C=C
1414, 1398	O-H bending
657, 641, 548	Zr-O-Zr
471, 466, 467	Zr-O

Diffuse Reflectance UV-Vis Spectra

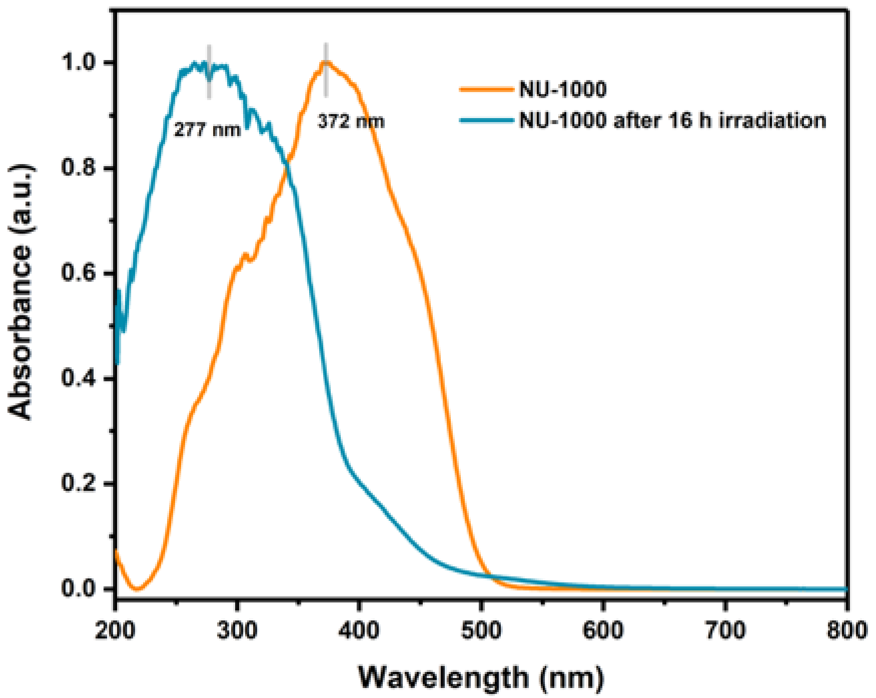

Figure S11: Solid state UV-Vis spectra of NU-1000 (in orange) and NU-1000 after 16 h irradiation (in blue). The solid-state UV-Vis spectra of NU-1000 before and after irradiation showed a significant change. The pristine NU-1000 has a maximum absorbance at 372 nm, while after irradiation the sample displays a hypsochromic shift, with the maximum absorbance centered at 277 nm.

Additional Photodegradation Experiments

Determination of the superoxide formation in TMPD photooxidation

To keep the concentration of TBAPy constant in reactions, MOF (2 mg) was digested in a mixture of D_2SO_4/DMSO-d_6 with TCE as internal standard. The concentration of ligand in MOF was calculated as 0.8 mmol/g.

N,N,N',N'-tetramethyl phenylenediamine (10 mg, 1 equiv.) was dissolved in a mixture of acetonitrile (2 mL) and DI water (2 mL). MOF (3.6 mg, 5 mol%) or H_4TBAPy (2 mg, 5 mol%) was used as photocatalyst. The reaction was irradiated with 390 nm LED in air and the product formation was monitored by UV-Vis spectroscopy.

NU-1000 irradiation in the presence of TMPD

Activated NU-1000 (3.6 mg) and TMPD (5.4 mg, 20 eq.) were added in a mixture of acetonitrile (2.5 mL) and DI water (1.25 µL). The reaction mixture was irradiated and stirred overnight under 390 nm at 33 °C, under O_2. After reaction, the solid residue was collected via centrifugation and dried at 80 °C. For NMR study, the solid powder was digested with in a mixture of D_2SO_4/d_6-DMSO, forming a clear solution.

NU-1000 irradiation in the presence of DABCO

Activated NU-1000 (10 mg, 4.59 µmol) and DABCO (5.1 mg, 10 eq.) were added in a mixture of acetonitrile (2.5 mL) and DI water (1.25 µL). The reaction mixture was irradiated and stirred overnight under 390 nm at 33 °C, under O_2. After reaction, the solid residue was collected via centrifugation and dried at 80°C. For NMR study, the solid powder was digested with in a mixture of D_2SO_4/d_6-DMSO, forming a clear solution.

NU-1000 irradiation in the presence of TEMPO

Activated NU-1000 (10 mg, 4.59 µmol) and TEMPO (7.2 mg, 10 eq.) were added in a mixture of acetonitrile (2.5 mL) and DI water (1.25 µL). The reaction mixture was irradiated and stirred overnight under 390 nm at 33 °C, under O_2. After reaction, the solid residue was collected via centrifugation and dried at 80 °C. For NMR study, the solid powder was digested with in a mixture of D_2SO_4/d_6-DMSO, forming a clear solution.

NU-1000 irradiation in the presence of triethylamine

Activated NU-1000 (10 mg, 4.59 µmol) and triethylamine (6.4 µL, 10 eq.) were added in a mixture of acetonitrile (2.5 mL) and DI water (1.25 µL). The reaction mixture was irradiated and stirred overnight under 390 nm at 33 °C, under O_2. After reaction, the solid residue was collected via centrifugation and dried at 80 °C. For NMR study, the solid powder was digested with in a mixture of D_2SO_4/d_6-DMSO, forming a clear solution.

NU-1000 irradiation in the presence of 1,5-dihydroxylnaphthalene

Activated NU-1000 (3.6 mg) and 1,5-dihydroxylnaphthalene (5 mg, 10 eq.) were added in a mixture of acetonitrile (2.5 mL) and DI water (1.25 µL). The reaction mixture was irradiated and stirred overnight under 390 nm at 33 °C, under O_2. After reaction, the solid residue was collected via centrifugation and dried at 80 °C. For NMR study, the solid powder was digested with in a mixture of D_2SO_4/d_6-DMSO, forming a clear solution.

NU-1000 irradiation in methanol

Activated NU-1000 (10 mg, 4.59 µmol) was added in a mixture of methanol (2.5 mL) and DI water (1.25 µL). The reaction mixture was irradiated and stirred overnight under 390 nm at 30 °C, under O_2. After reaction, the solid residue was collected via centrifugation and dried at 80 °C. For NMR study, the solid powder was digested with in a mixture of D_2SO_4/d_6-DMSO, forming a clear solution.

NU-1000 irradiation in the presence styrene

Activated NU-1000 (20 mg) and styrene (21.5 µL, 20 eq.) were added in acetonitrile (5 mL). The reaction mixture was irradiated and stirred overnight under 390 nm at 33 °C, under O_2. After reaction, the solid residue was collected via centrifugation and dried at 80 °C. For NMR study, the solid powder was digested with in a mixture of D_2SO_4/d_6-DMSO, forming a clear solution.

NU-1000 irradiation in the presence of HEPES

Activated NU-1000 (3.6 mg) and HEPES (4 mg, 10 eq.) were added in DI H_2O (2 mL). The reaction mixture was irradiated and stirred overnight under 390 nm at 33 °C, under O_2. After reaction, the solid residue was collected via centrifugation and dried at 80 °C. For NMR study, the solid powder was digested with in a mixture of D_2SO_4/d_6-DMSO, forming a clear solution.

Table S4: Additional reaction conditions for the photodegradation of NU-1000.

Entry	Deviation from the standard conditions	Conversion (%)
**S1**	TMPD (20 eq.)	4
**S2**	DABCO (10 eq.)	0
**S3**	TEMPO (10 eq.)	0
**S4**	MeOH as solvent	~44*
**S5**	Triethylamine (10 eq.)	0
**S6**	1,5-Dihydroxylnaphthalene (10 eq.)	0
**S7**	Styrene (20 eq.)	100
**S8**	HEPES (10 eq.)	0

*yields a complex mixture of aromatic products as shown in Figure S34.

Photodegradation of NU-1000 dry powder

NU-1000 powder was spread onto a glass slide and irradiated with 390 nm light for four days. A sample of the irradiated MOF was digested for ^{1}H NMR analysis, revealing approximately 34 % degradation (**Figure S12**).

TGA analysis was conducted on the sample and compared with pristine NU-1000, as shown in **Figure S13**.

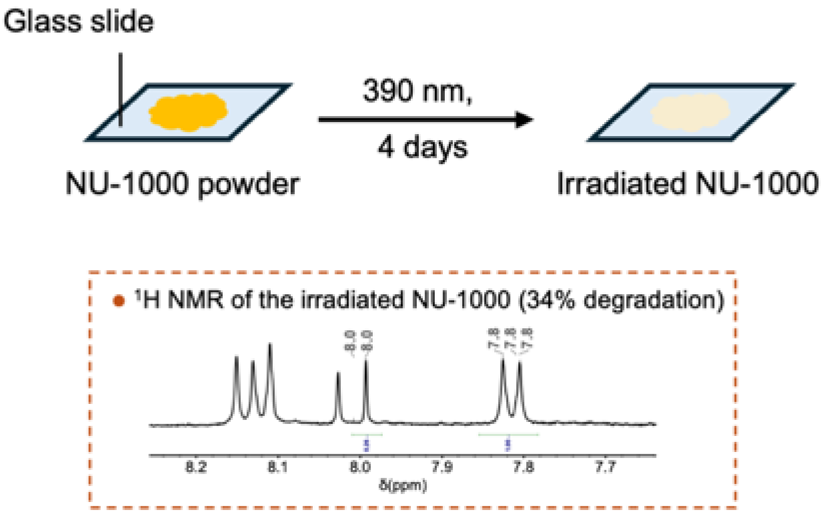

Figure S12: Illustration of photodegradation experiment conducted on dry NU-1000 powder and ^{1}H-NMR spectrum of the irradiated sample.

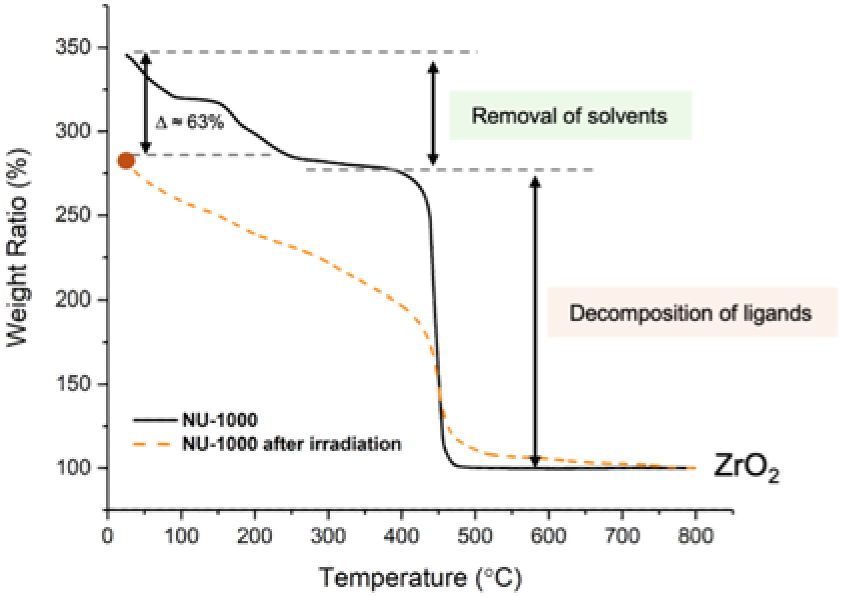

Figure S13: TGA plots of pristine NU-1000 and irradiated NU-1000.

Calculation of the TBAPy concentration in NU-1000

NU-1000, formula – $C_{88}H_{44}O_{32}Zr_6$, Z = 3, Cell volume V = 22122.8 Å^3 = 2.21228 $\cdot\ 10^{-20}\ cm^3$

$$c(\mathrm{TBAPy}) = \frac{n}{V} = \frac{N}{N_\mathrm{A}\cdot V} = \frac{6}{6.022\cdot 10^{23}\cdot 2.21228\cdot 10^{-20}}\ mol/ml$$

$$c(\mathrm{TBAPy}) = 0.448\cdot mol/l$$

Estimation of the NU-1000 degradation time under ambient light

The power density of the sun's irradiation on the surface of the earth is around 1.4 kW/m^2.[5] Since at the Earth's surface approximately 5% of the radiation is in the ultraviolet region (300-400 nm),[6] the energy density from UV region can be estimated to be around 70 W/m^2. Given by the power density provided by 390 nm LED in this experiment, the estimated degradation time of NU-1000 can be calculated about 105 hours (in suspension), and 83 days (in solid state). However, we note that the degradation rate of the dry powder is highly dependent on its dispersion and form factor, meaning reaction rates may vary significantly.

Table S5: Estimated time for the photodegradation of NU-1000 under sun irradiation.

Entry	Power density (W/m^2)	Estimated photodegradation (in %) time
**NU-1000 (in MeCN suspension)**	490 (LED lamp)	18 hours for 100 % (see Entry 1 Table 1)
	70 (estimated sun irradiation)	126 hours for 100% (estimated value)
**NU-1000 (powder)**	490 (LED lamp)	4 days for 34 % degradation (See SI Figure S12)
	70 (estimated sun irradiation)	83 days for 100% (estimated value)

Characterization of the NU-1000 photodegradation product

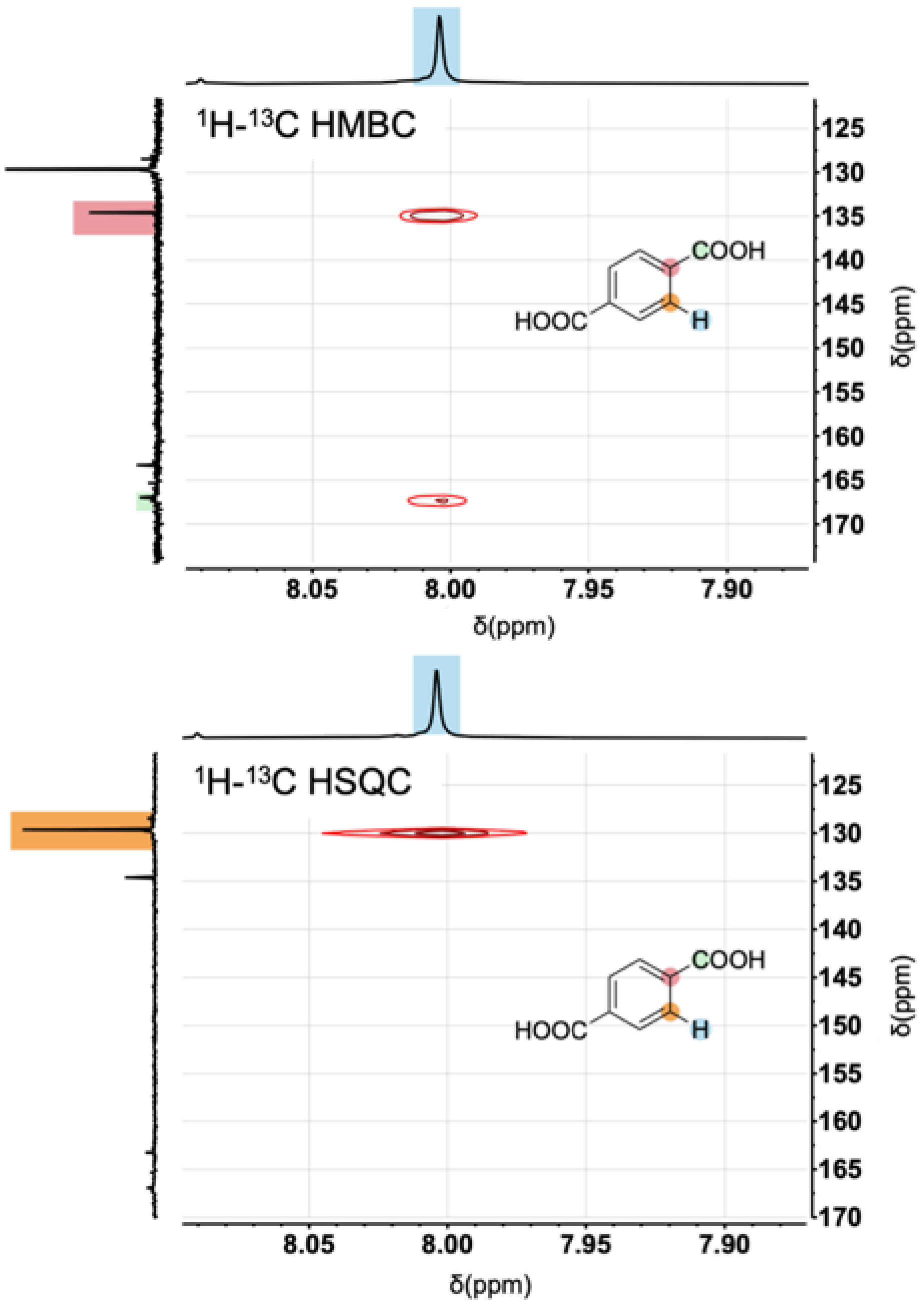

Figure S14: ^{1}H-^{13}C HMBC and ^{1}H-^{13}C HSQC spectra (DMSO-d_6) of the product after NU-1000 photodegradation

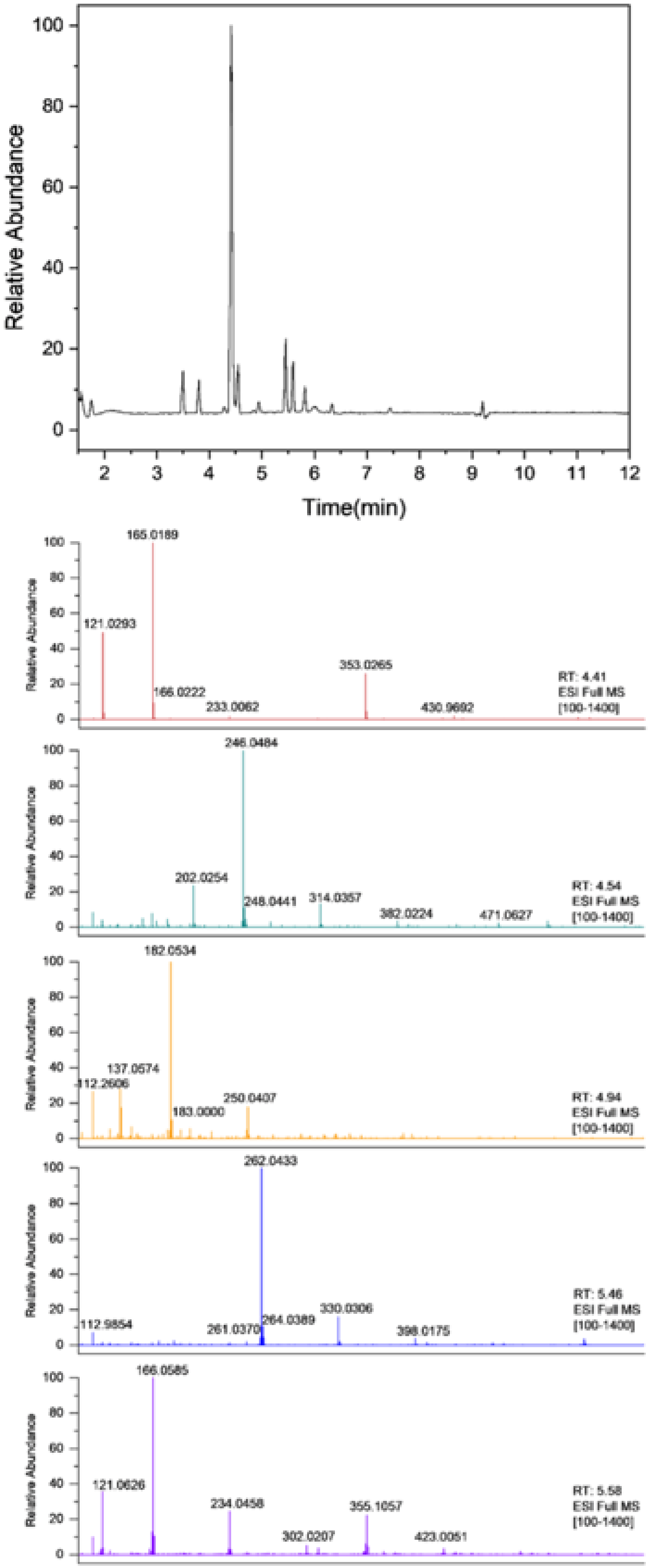

Figure S15: High resolution ESI-MS spectrum of the crude product (digested in D_2SO_4) after NU-1000 degradation.

Characterization data from photodegradation experiment conducted at the University of Vienna

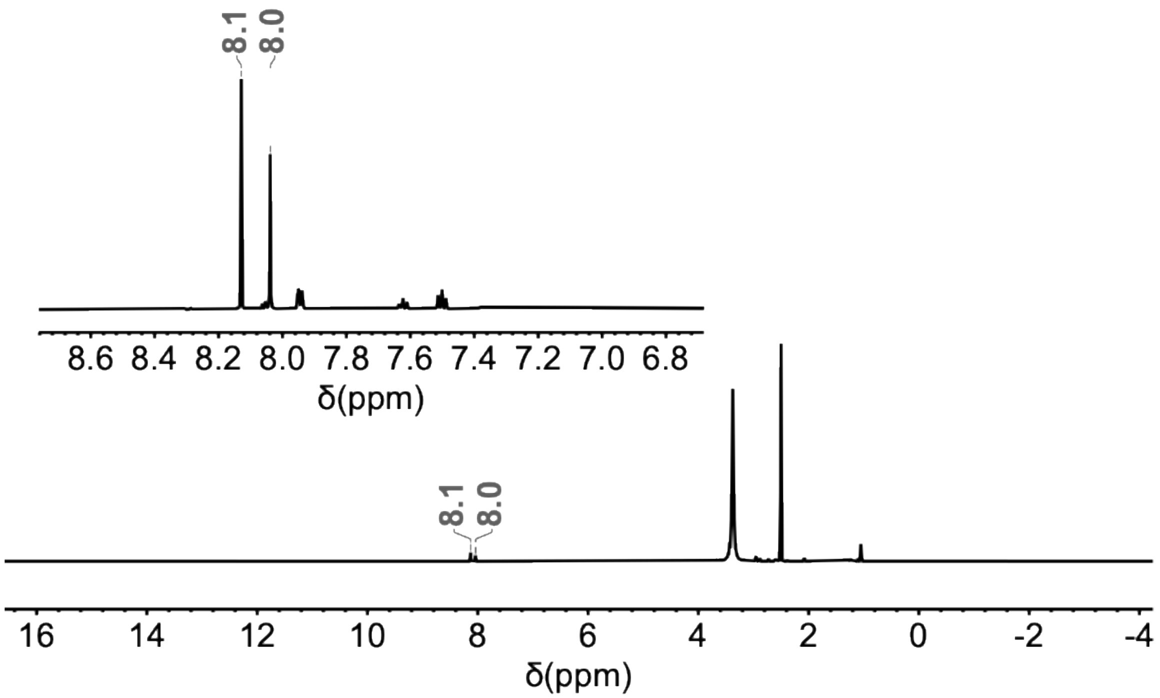

Figure S16: ^{1}H-NMR (DMSO-d_6, 500 MHz) of the crude product after photodegradation with NU-1000 (20 mg, 9.19 µmol), acetonitrile (5 mL), DI H_2O (2.5 µL), 427 nm, O_2, 18 h. Benzoic acid appeared as the impurity from the starting material, DMSO solvent residual peak shows at 2.5 ppm and water at 3.3 ppm. Inset: zoom in part for the proton of terephthalic acid (8.0 ppm) and formic acid (8.1 ppm).

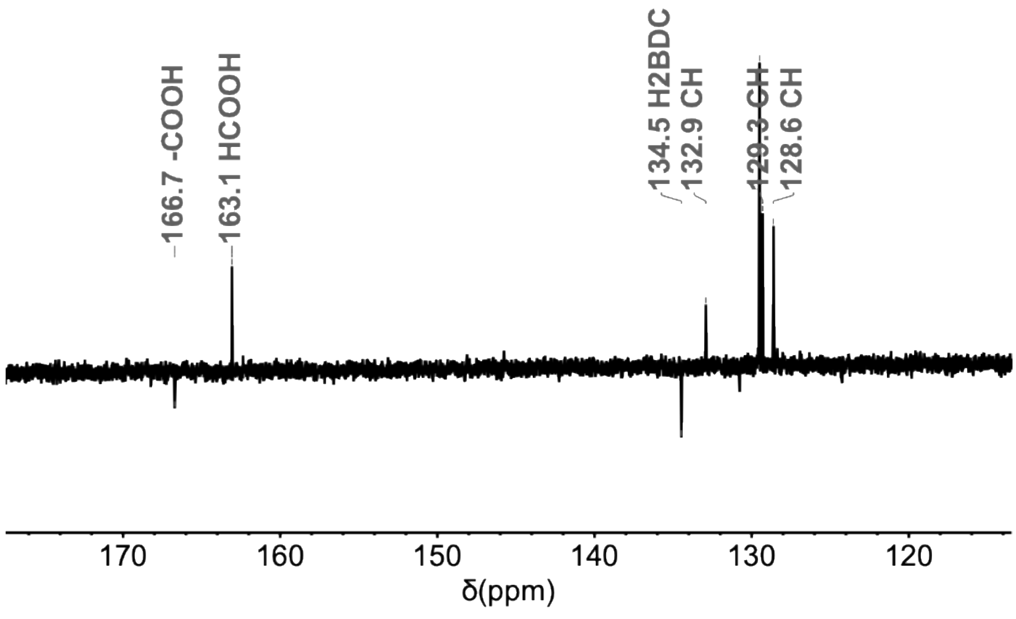

Figure S17: ^{13}C-NMR (DMSO-d_6, 500 MHz) of the crude product after photodegradation with NU-1000 (20 mg, 9.19 µmol), acetonitrile (5 mL), DI H_2O (2.5 µL), 30 °C, 427 nm, O_2, 18 h.

NMR Spectra of Photodegradation Experiments

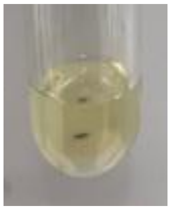
(reaction after 18 h in DMSO)

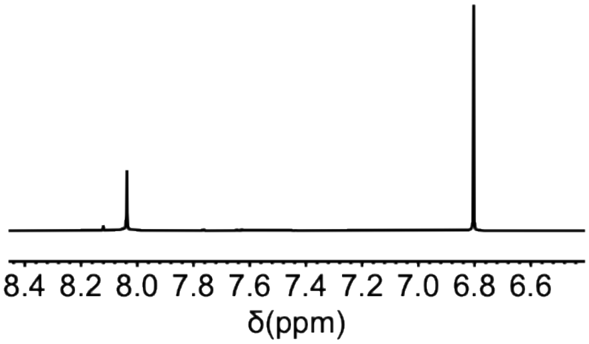

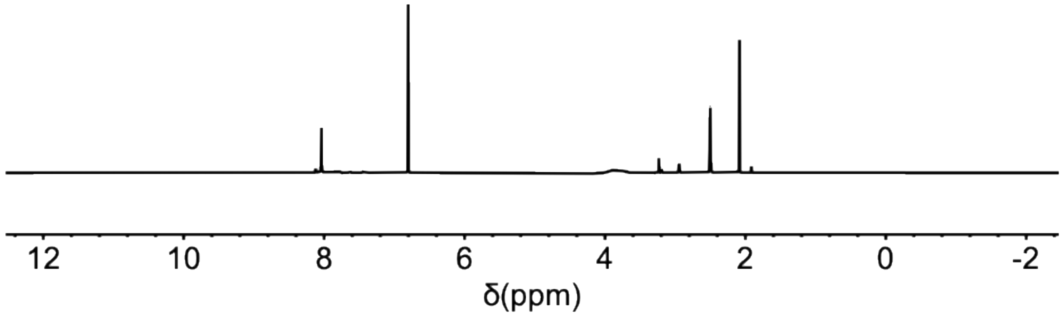

Figure S18: ^{1}H-NMR (DMSO-d$_6$, 500 MHz) of the crude product after 18 hours photodegradation with NU-1000 in DMSO-d$_6$. The inset photography showed all solid part was fully dissolved in DMSO, forming a clear solution.

Calculation of the degradation extent in Table 1

$$\text{Conversion of } \mathrm{H_2BDC}\ (\%) = \frac{n(\mathrm{H_2BDC})}{[n(\mathrm{H_2BDC}) + n(TBAPy)]} \times 100\%$$

$$= \frac{\frac{1}{4} n_H(\mathrm{H_2BDC})}{\left[\frac{1}{4} n_H(\mathrm{H_2BDC}) + \frac{1}{8} n_H(TBAPy)\right]} \times 100\%$$

$$= \frac{\frac{1}{4} \int n_H\ (\mathrm{H_2BDC})}{\left[\frac{1}{4} \int n_H\ (\mathrm{H_2BDC}) + \frac{1}{8} \int n_H\ (TBAPy)\right]} \times 100\%$$

$$Set \int n_H\ (TBAPy) = 1$$

$$\text{Conversion of } \mathrm{H_2BDC}\ (\%) = \frac{2 \int n_H\ (\mathrm{H_2BDC})}{2 \int n_H\ (\mathrm{H_2BDC}) + 1} \times 100\%$$

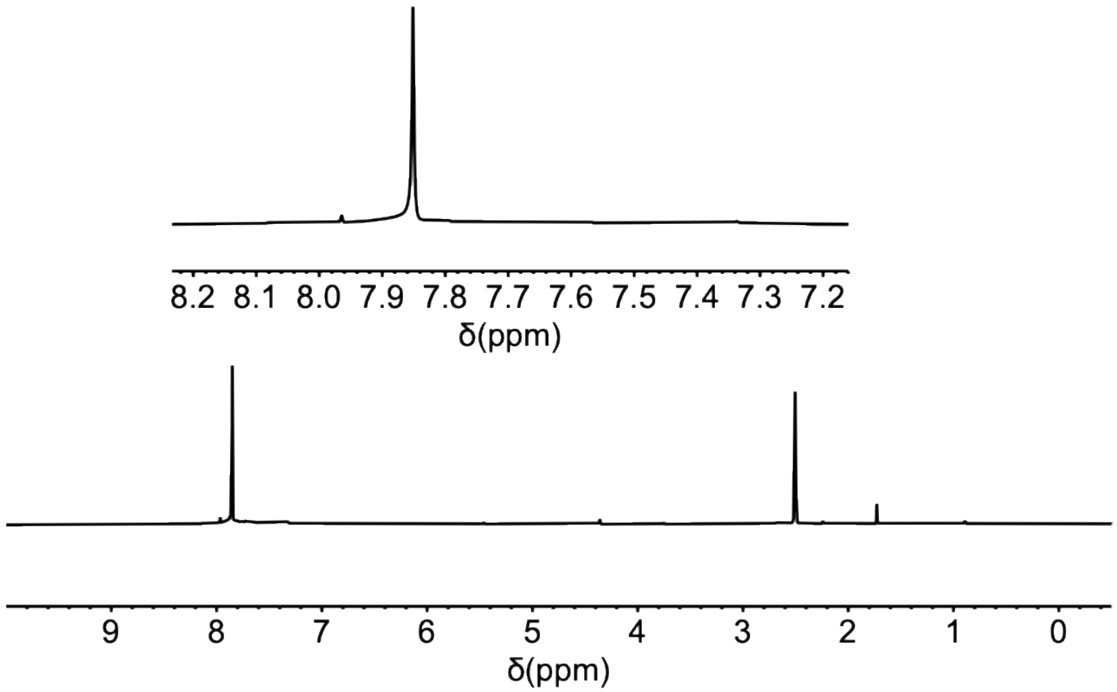

Figure S19: ^{1}H-NMR (DMSO-d6, 500 MHz) of the crude product after photodegradation with NU-1000 (20 mg, 9.19 µmol), acetonitrile (5 mL), DI H_2O (2.5 µL), 33 °C, 390 nm, O_2, 18 h. (Entry 1)

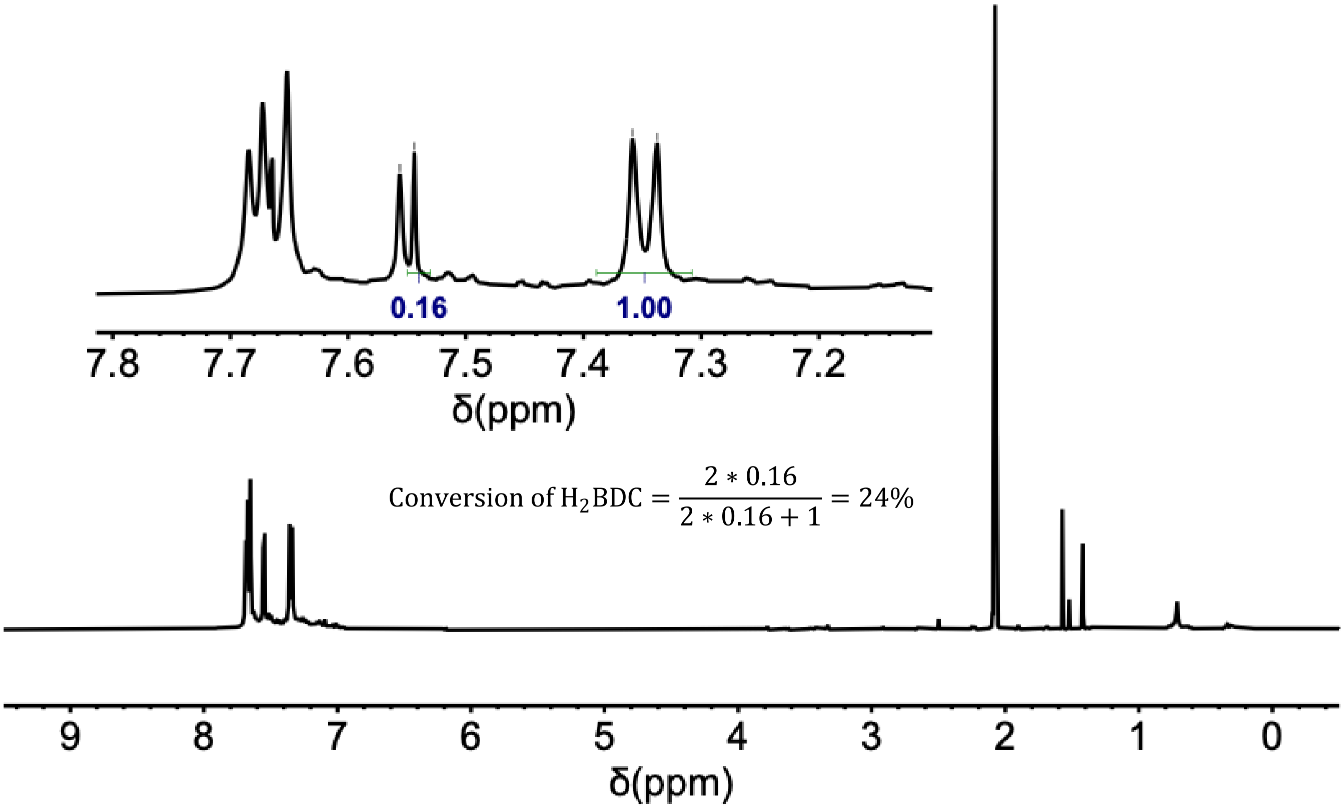

Figure S20: ^{1}H-NMR (DMSO-d6, 500 MHz) of the crude product after photodegradation with NU-1000 (20 mg, 9.19 µmol), anhydrous acetonitrile (5 mL), 33 °C, 390 nm, O_2, 18 h. (Entry 2)

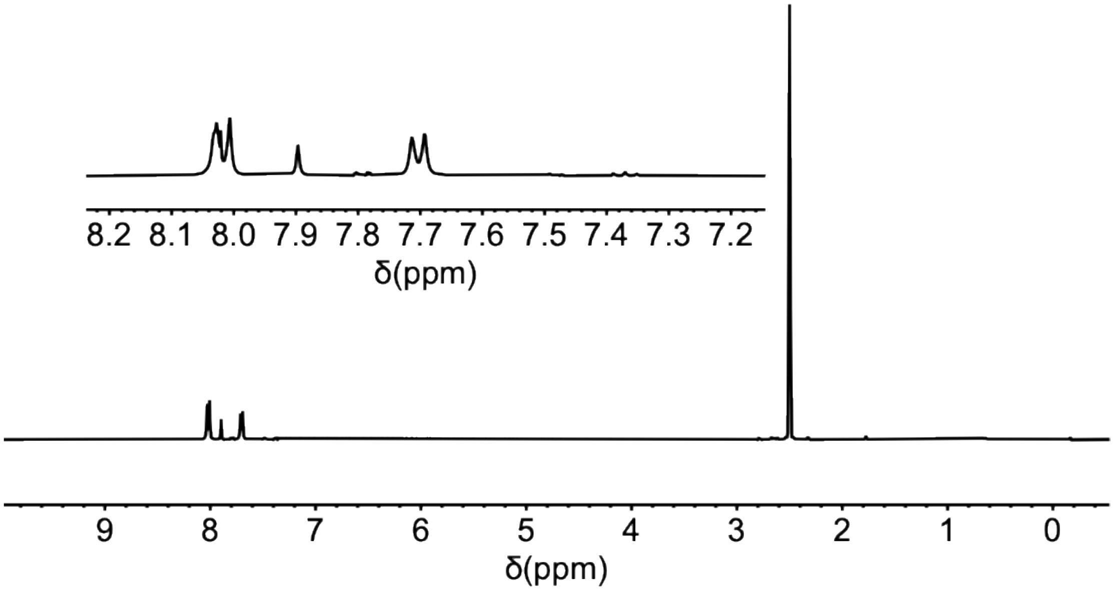

Figure S21: ^{1}H-NMR (d_6-DMSO, 500 MHz) of the crude product after photodegradation with NU-1000 (20 mg, 9.19 µmol), acetonitrile (5 mL), DI H_2O (2.5 µL), 33 °C, dark, O_2, 18 h. (Entry 3)

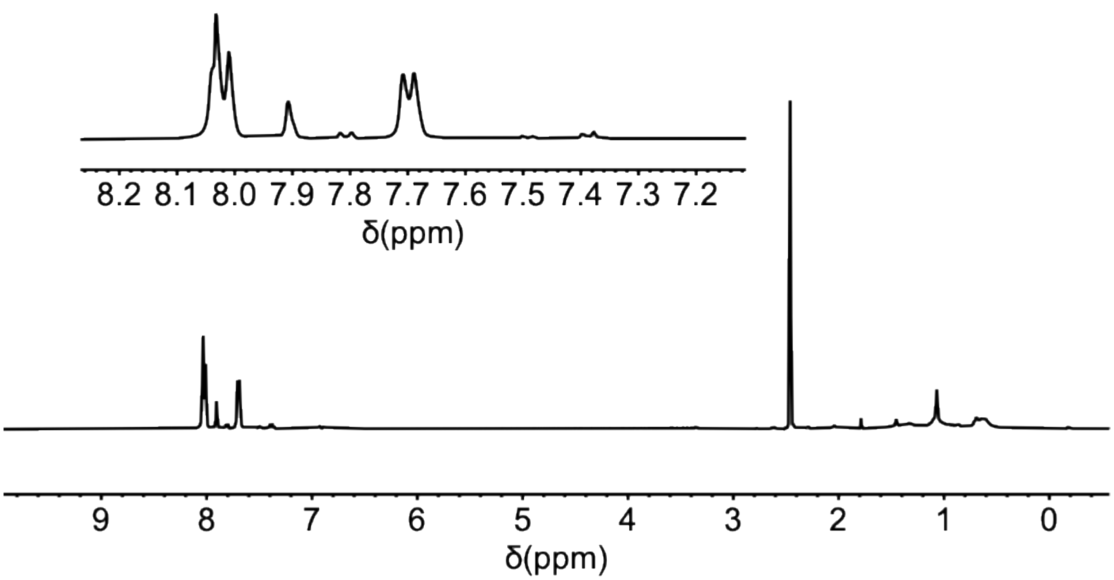

Figure S22: ^{1}H-NMR (d_6-DMSO, 500 MHz) of the crude product after photodegradation with NU-1000 (20 mg, 9.19 µmol), acetonitrile (5 mL), DI H_2O (2.5 µL), 33 °C, 390 nm, Ar, 18 h. (Entry 4)

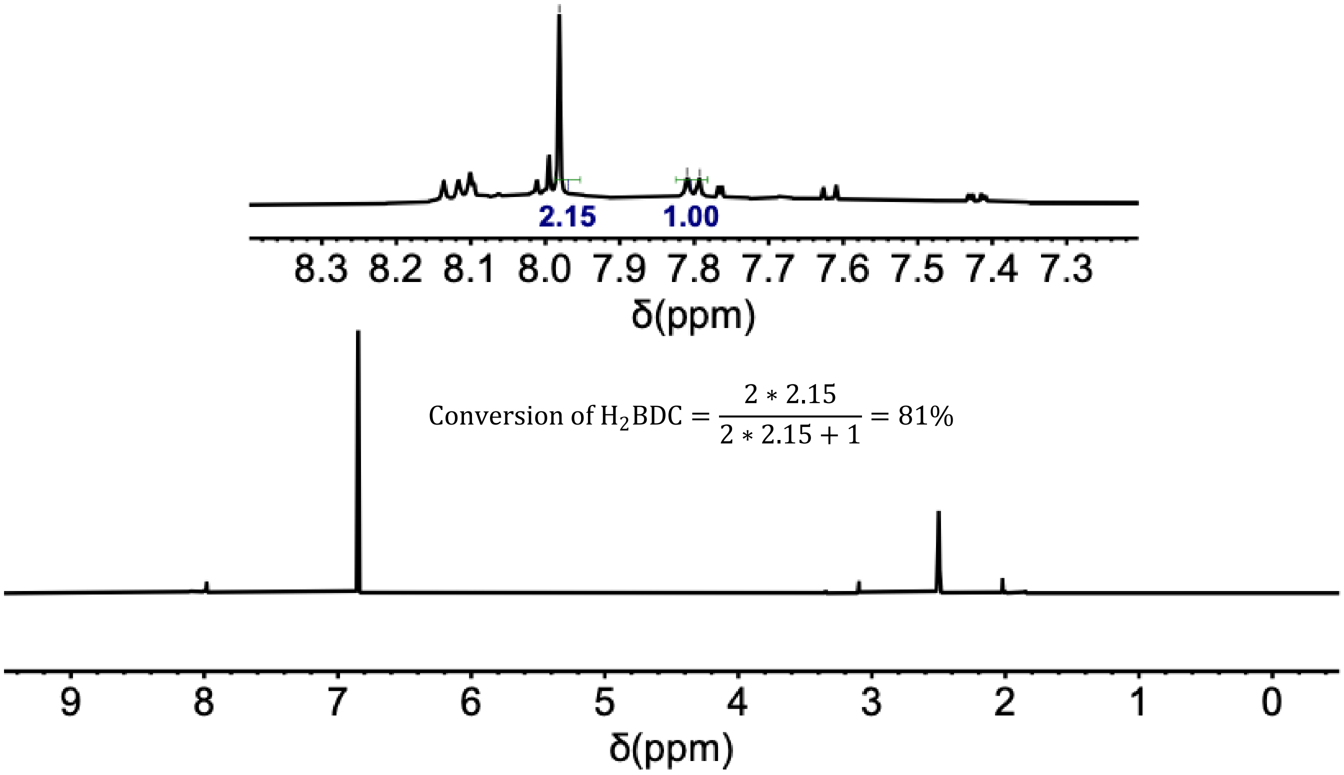

Figure S23: ^{1}H-NMR (d_6-DMSO, 500 MHz) of the crude product after photodegradation with NU-1000 (20 mg, 9.19 µmol), acetonitrile (5 mL), DI H_2O (2.5 µL), 33 °C, 390 nm, Air, 18 h. (Entry 5)

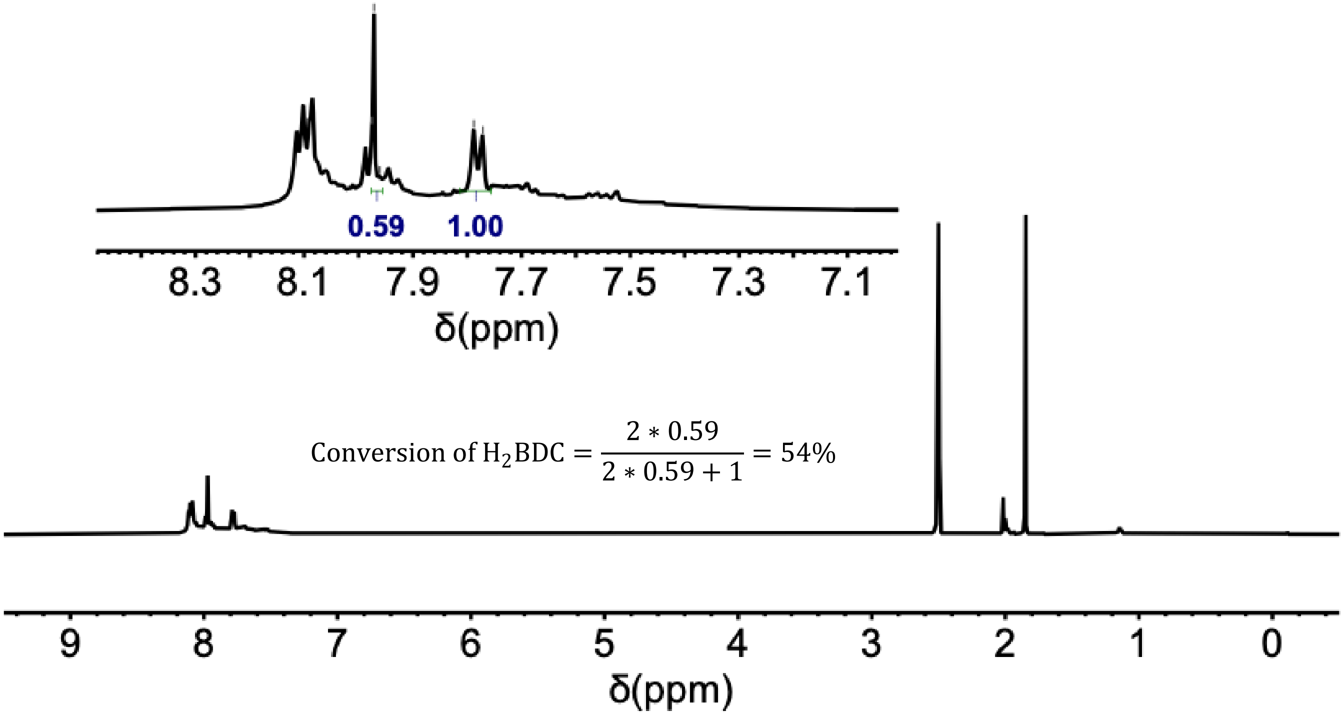

Figure S24: ^{1}H-NMR (d_6-DMSO, 500 MHz) of the crude product after photodegradation with NU-1000 (20 mg, 9.19 µmol), acetic acid (20 µl, 5 eq.), acetonitrile (5 mL), 33 °C, 390 nm, O_2, 18 h. (Entry 6)

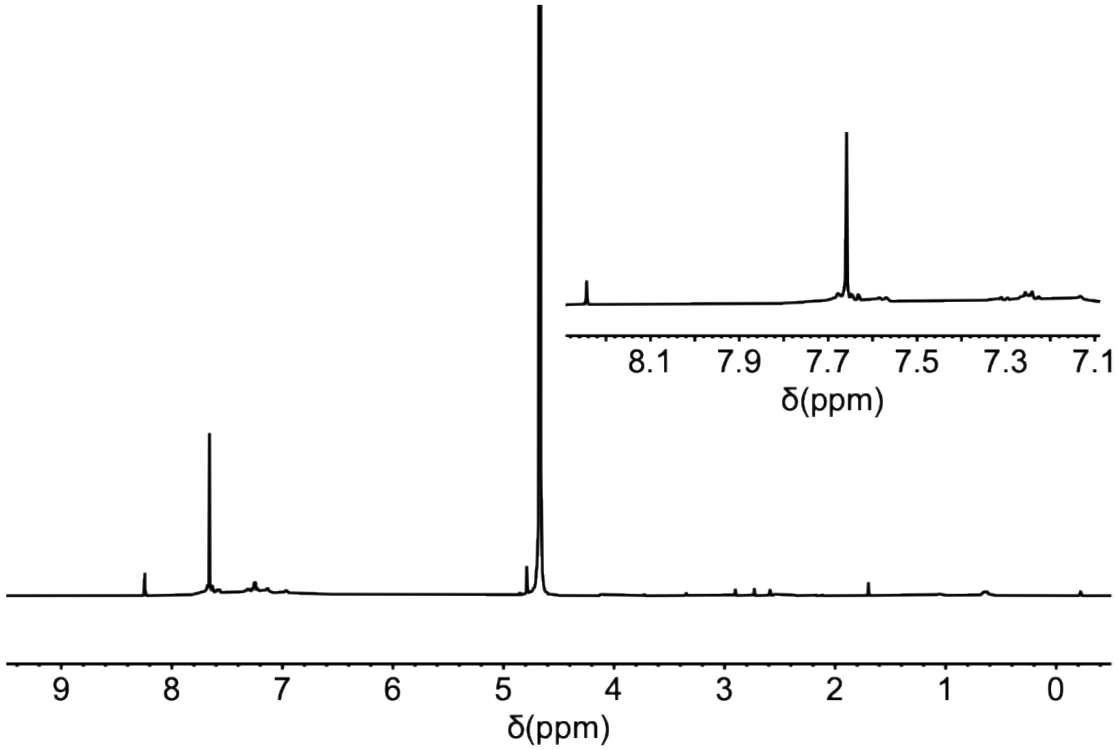

Figure S25: ^{1}H-NMR (D_2O, 500 MHz) of the crude product after photodegradation with NU-1000 (20 mg, 9.19 µmol), DI H_2O (5 mL), 30 °C, 390 nm, O_2, 18 h. (Entry 7)

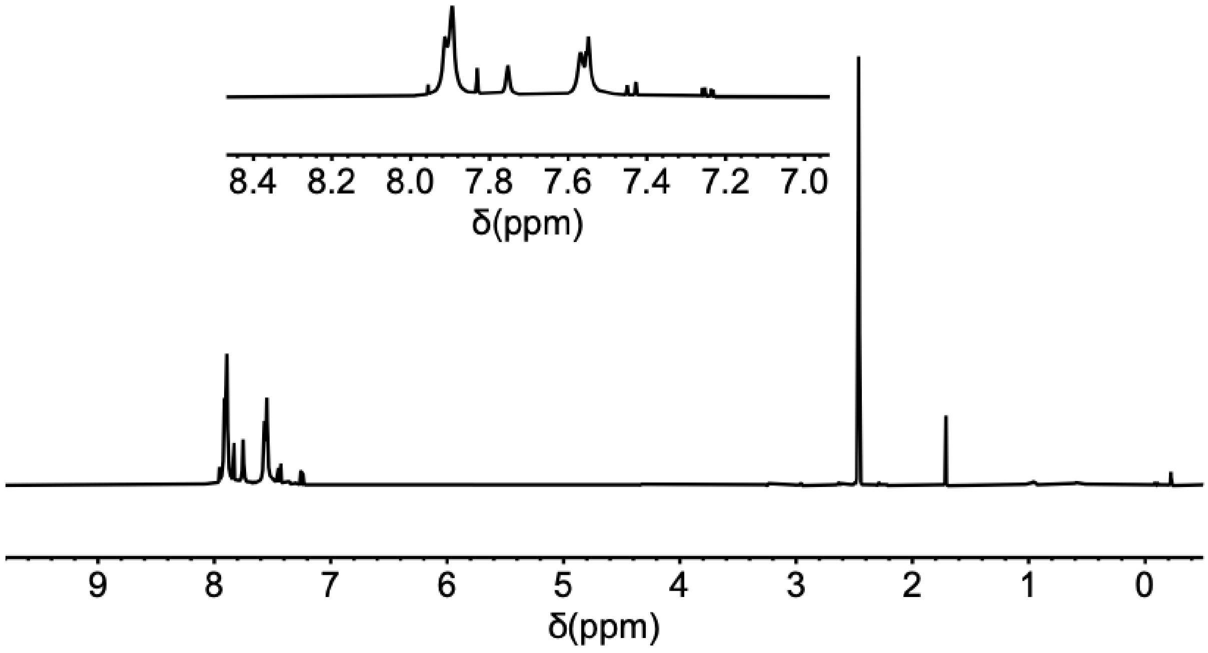

Figure S26: ^{1}H-NMR (d_6-DMSO, 500 MHz) of the crude product after photodegradation with NU-1000 (20 mg, 9.19 µmol), DI H_2O (5 mL), 30 °C, 390 nm, Ar, 18 h. (Entry 8)

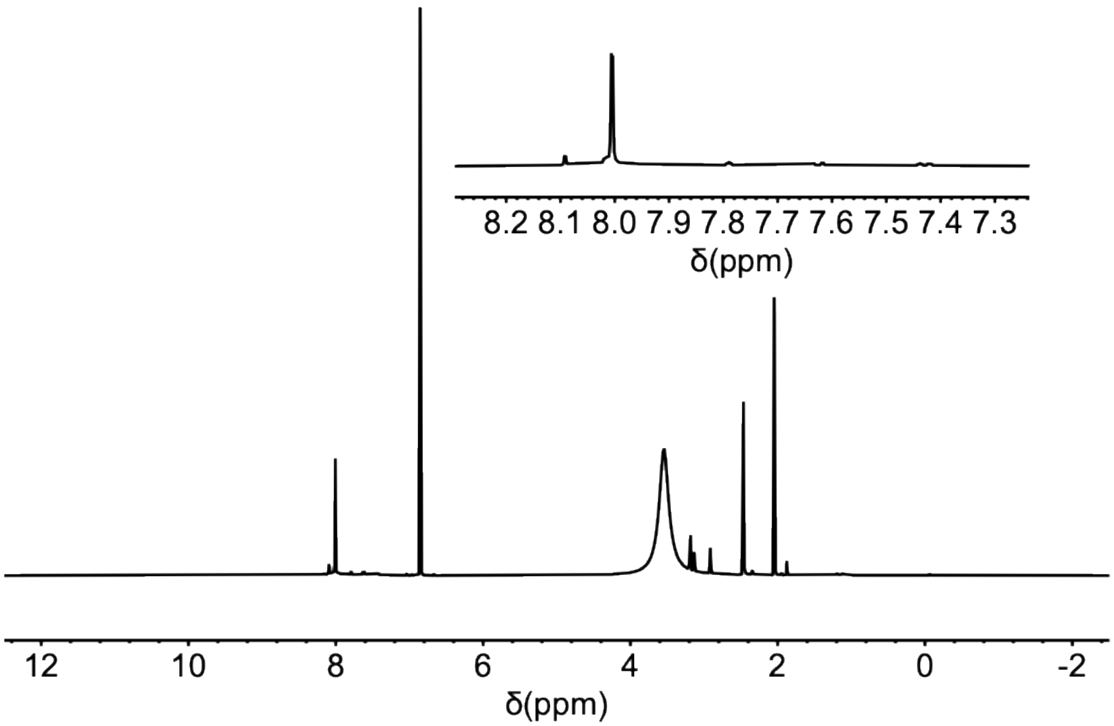

Figure S27: ^{1}H-NMR (d_6-DMSO, 500 MHz) of the crude product after photodegradation with NU-1000 (20 mg, 9.19 µmol), DMSO (5 mL), DI H_2O (2.5 µL), 30 °C, 390 nm, O_2, 18 h. (Entry 9)

$$\text{Conversion of } H_2BDC = \frac{2 * 0.04}{2 * 0.04 + 1} = 7\%$$

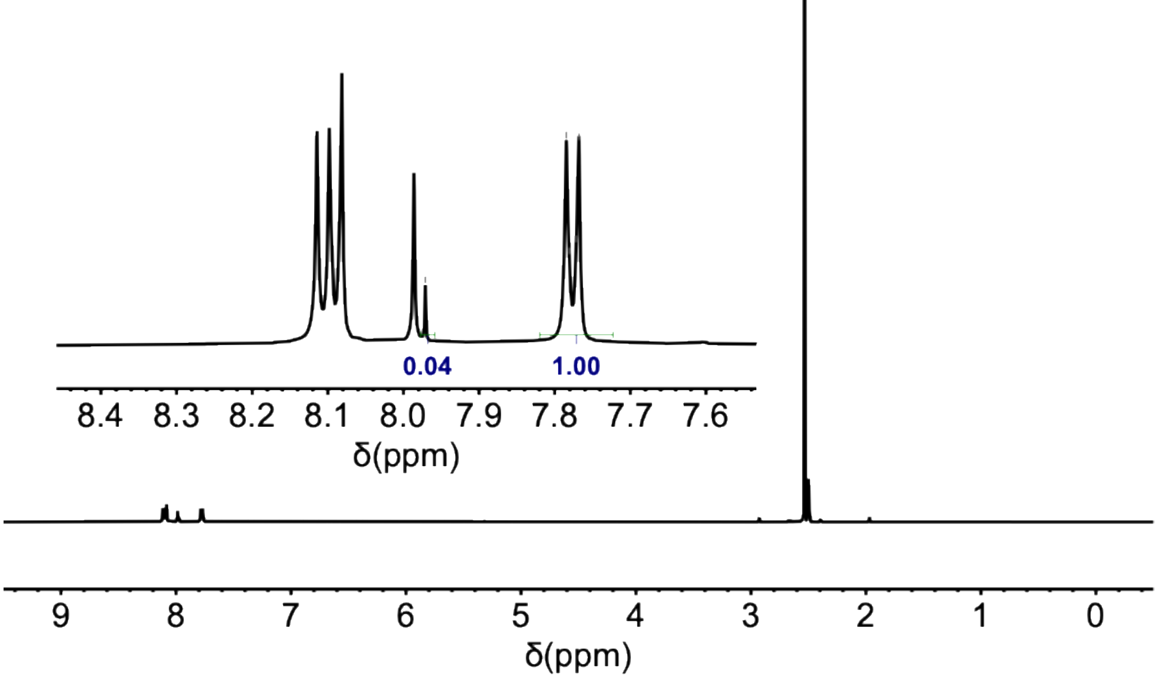

Figure S28: ^{1}H-NMR (d_6-DMSO, 500 MHz) of the crude product after photodegradation with NU-1000 (20 mg, 9.19 µmol), anhydrous DMSO (5 mL), 30 °C, 390 nm, O_2, 18 h. (Entry 10)

$$\text{Conversion of } H_2BDC = \frac{2 * 0.23}{2 * 0.23 + 1} = 32\%$$

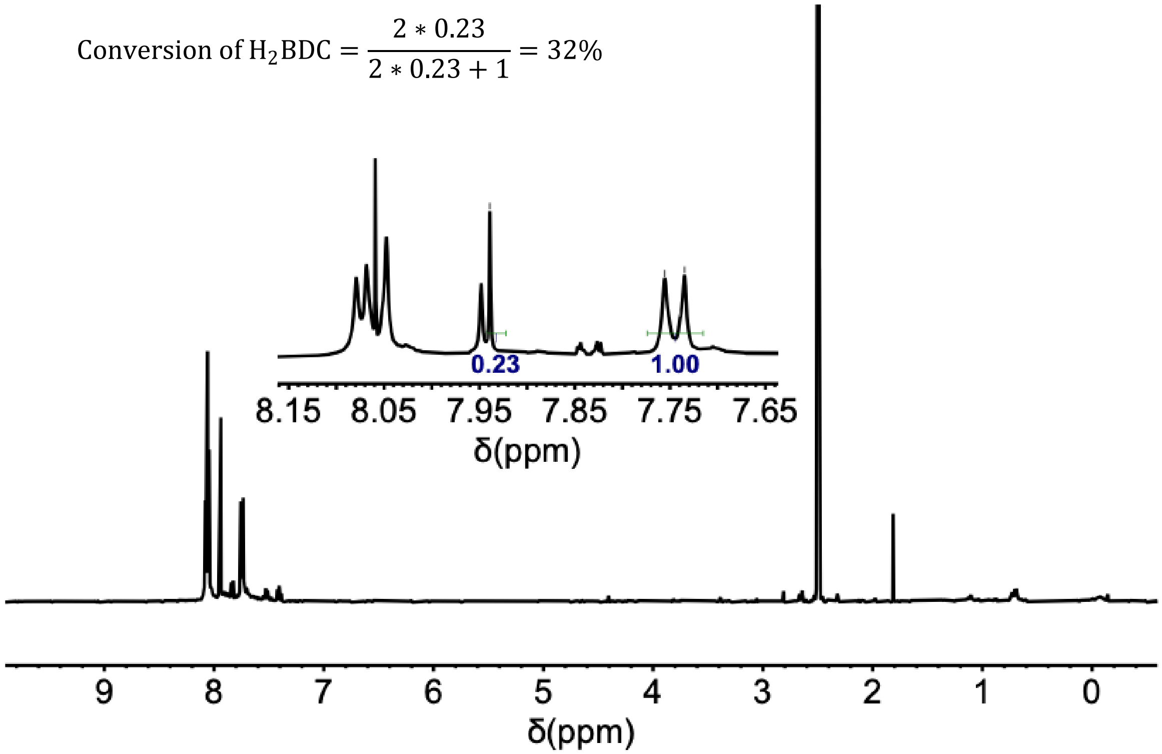

Figure S29: ^{1}H-NMR (d_6-DMSO, 500 MHz) of the crude product after photodegradation with NU-1000 (20 mg, 9.19 µmol), acetonitrile (5 mL), DI H_2O (2.5 µL), 33 °C, 440 nm, O_2, 18 h. (Benzoic acid appeared as the impurity from the starting material) (Entry 11)

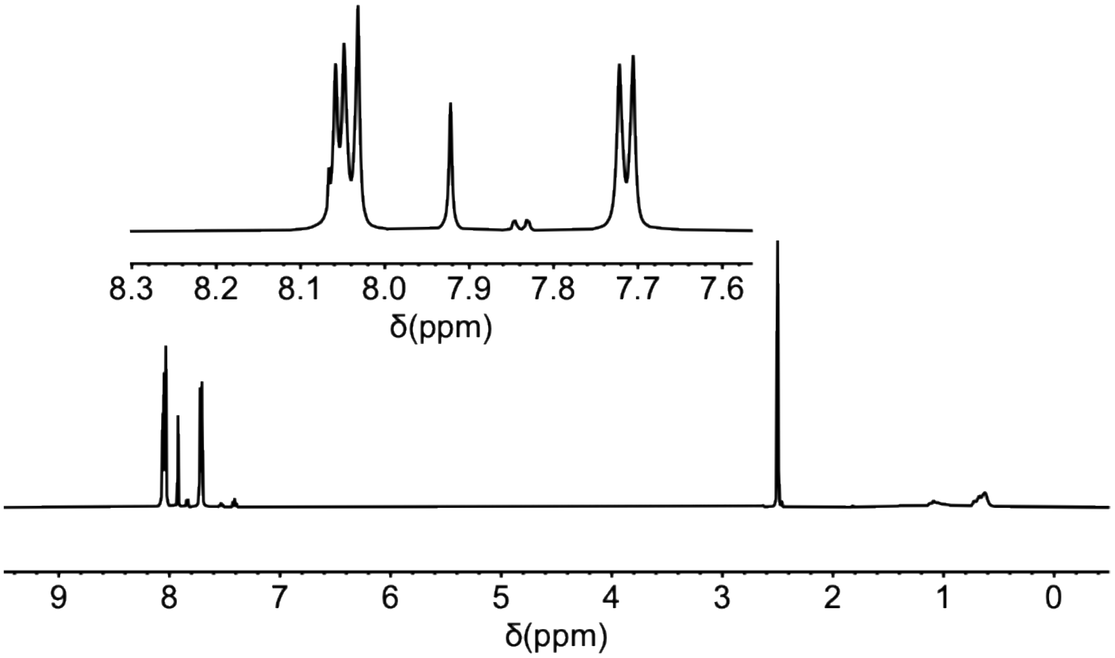

Figure S30: ^{1}H-NMR (d_6-DMSO, 500 MHz) of the crude product after photodegradation with NU-1000 (20 mg, 9.19 µmol), acetonitrile (5 mL), DI H_2O (2.5 µL), 33 °C, 525 nm, O_2, 18 h. (Benzoic acid appeared as the impurity from the starting material.) (Entry 12)

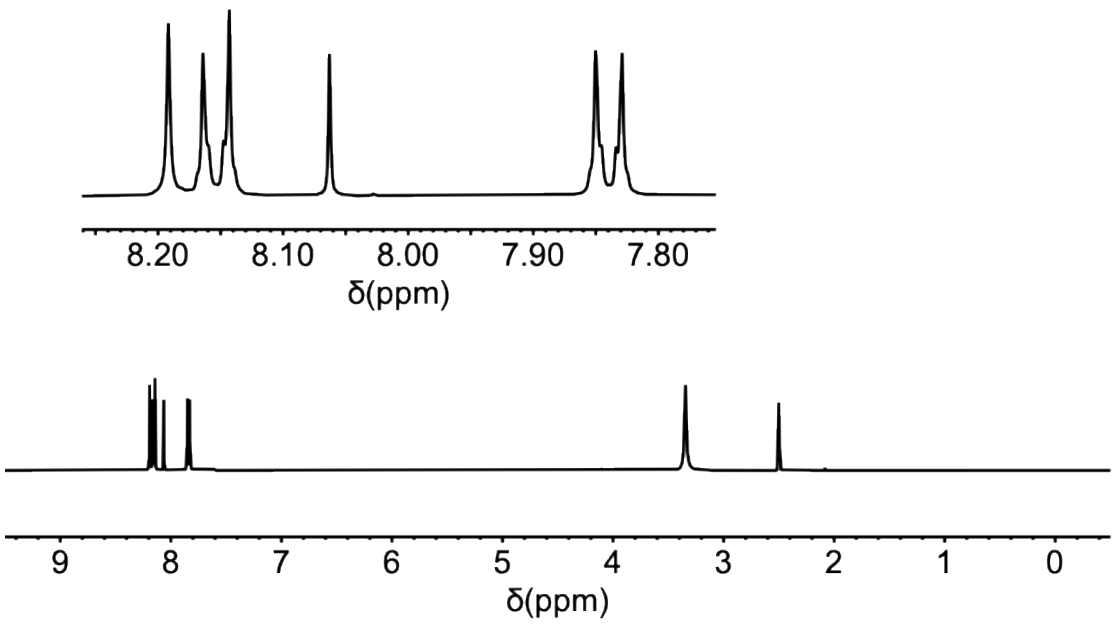

Figure S31: ^{1}H-NMR (d_6-DMSO, 500 MHz) of the crude product after photodegradation with H_4TBAPy (40 mg, 58.6 µmol), acetonitrile (5 mL), DI H_2O (2.5 µL), 33 °C, 390 nm, O_2, 18 h. (Entry 13)

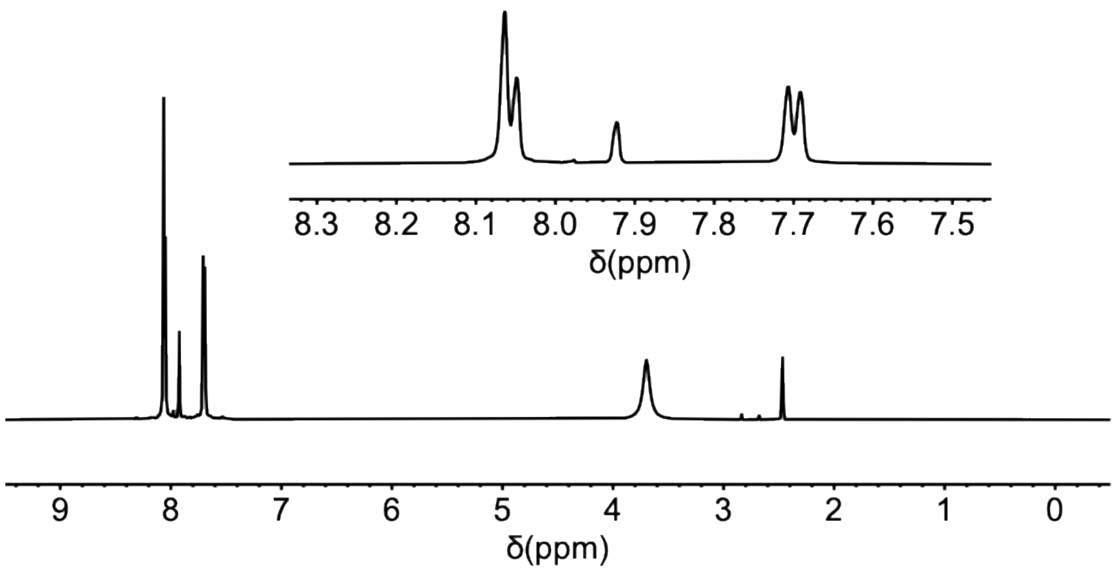

Figure S32: ^{1}H-NMR (d_6-DMSO, 500 MHz) of the crude product after photodegradation with H_4TBAPy (40 mg, 58.6 µmol), DMSO (5 mL), DI H_2O (2.5 µL), 30 °C, 390 nm, O_2, 18 h. (Entry 14)

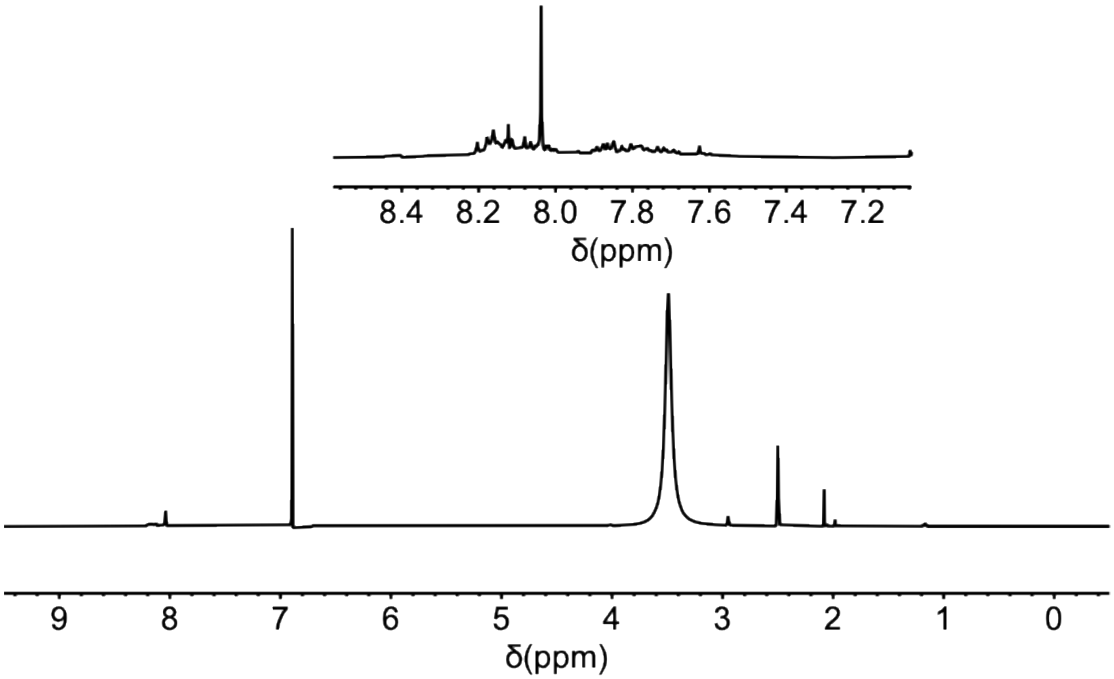

Figure S33: ^{1}H-NMR (d_6-DMSO, 500 MHz) of the crude product after photodegradation with H_4TBAPy (10 mg, 14.7 µmol), DMSO (5 mL), DI H_2O (2.5 µL), 30 °C, 390 nm, O_2, 18 h. (Entry 15)

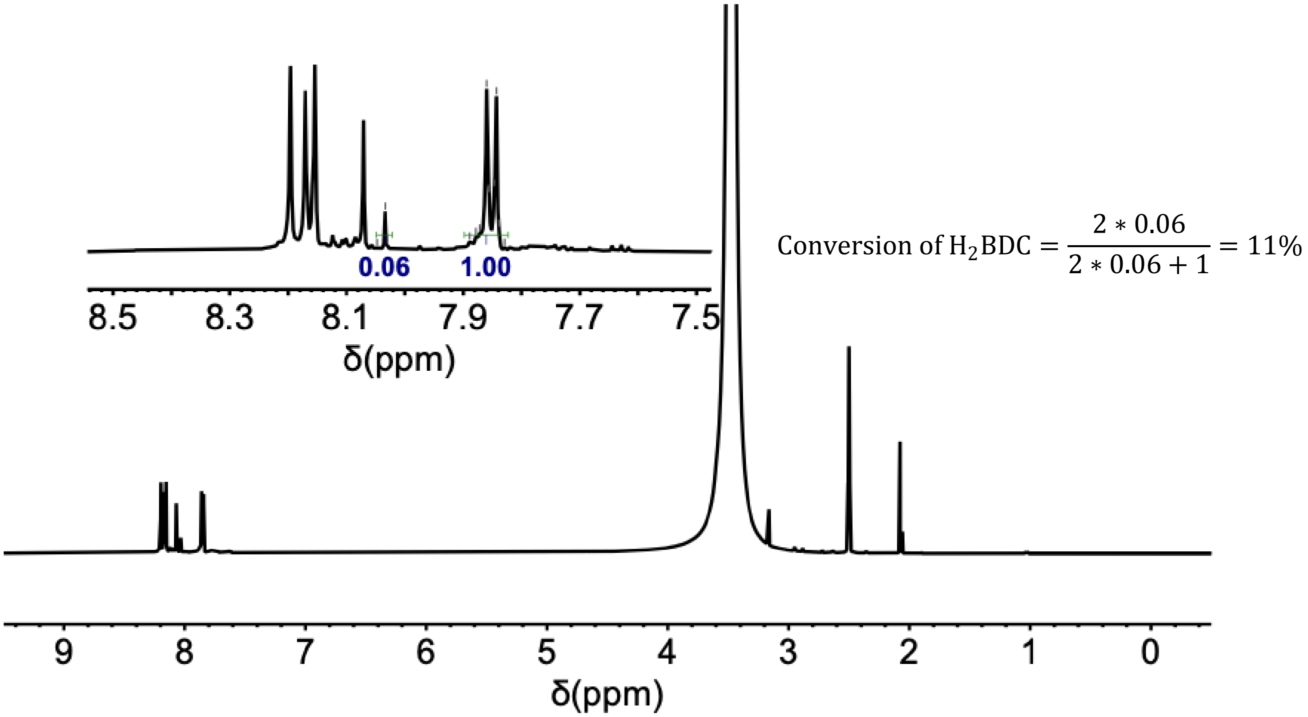

Figure S34: ^{1}H-NMR (d_6-DMSO, 500 MHz) of the crude product after photodegradation with H_4TBAPy (10 mg, 14.7 µmol), DMSO (5 mL), DI H_2O (2.5 µL), 30 °C, 440 nm, O_2, 18 h. (Entry 16)

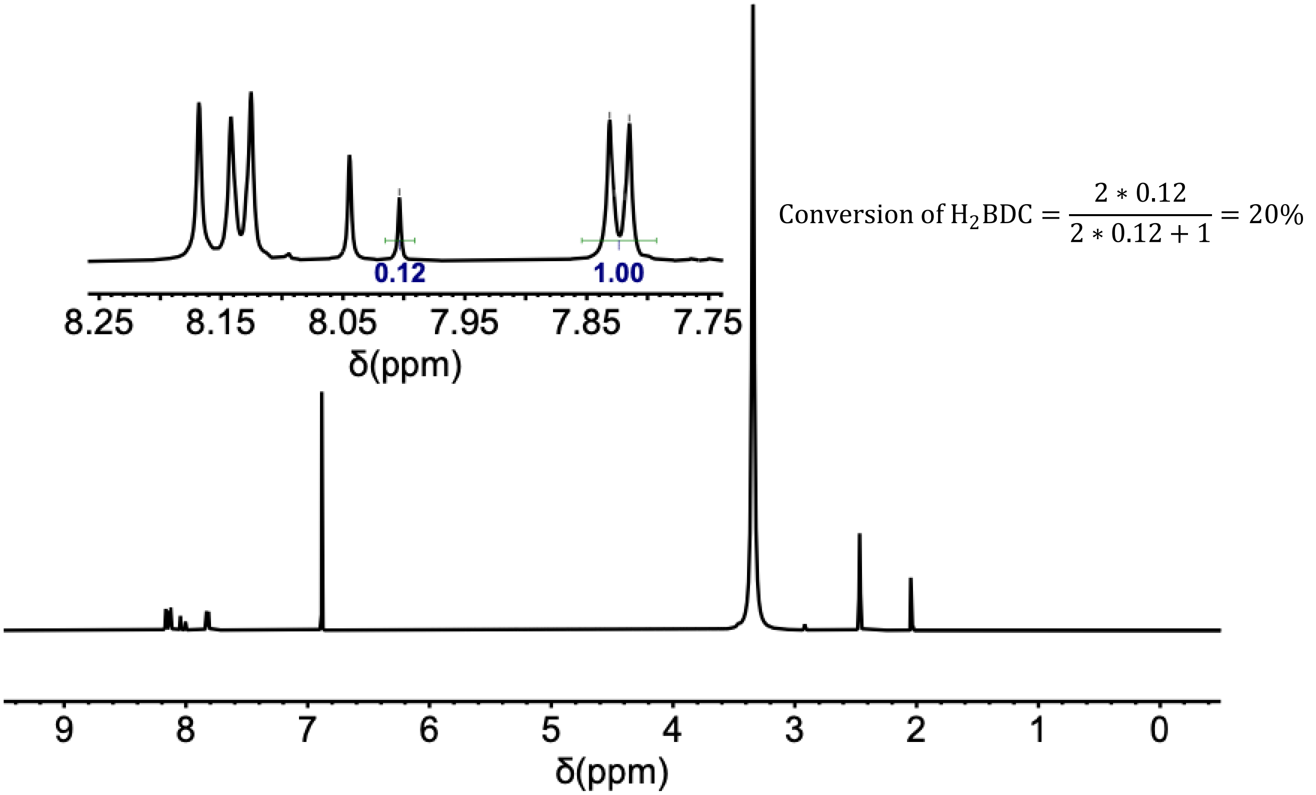

Figure S35: ^{1}H-NMR (d_6-DMSO, 500 MHz) of the crude product after photodegradation with H_4TBAPy (10 mg, 14.7 µmol), $ZrOCl_2·8H_2O$ (2 mg), DMSO (5 mL), 30 °C, 390 nm, O_2, 18 h. (Entry 17)

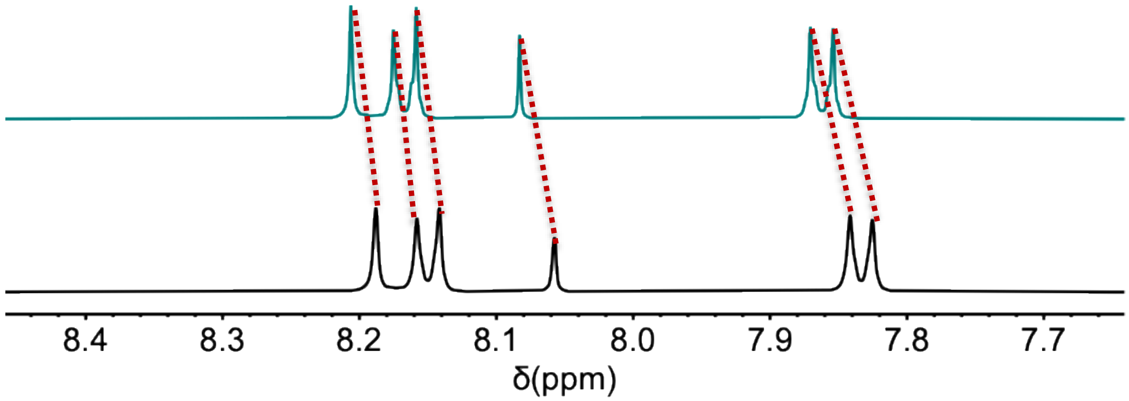

Figure S36: ^{1}H-NMR (d_6-DMSO, 500 MHz) of H_4TBAPy in different concentrations. (Top) H_4TBAPy in d_6-DMSO – 0.003 mol/L; (bottom) H_4TBAPy in d_6-DMSO – 0.01 mol/L

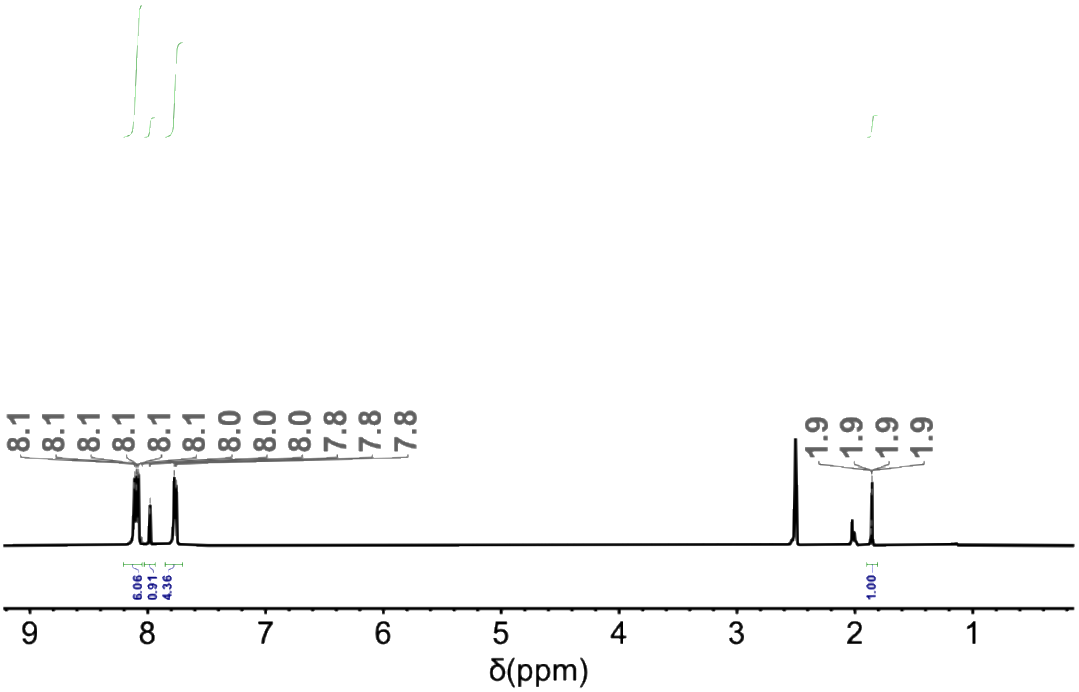

Figure S37: ^{1}H-NMR (d_6-DMSO, 500 MHz) of the crude product after reaction of NU-1000 (4 mg), acetic acid (4 µL, 5 eq.), acetonitrile (1 mL), 33 °C, 390 nm, 18 h. The integral shows the ratio between acetate and ligand TBAPy.

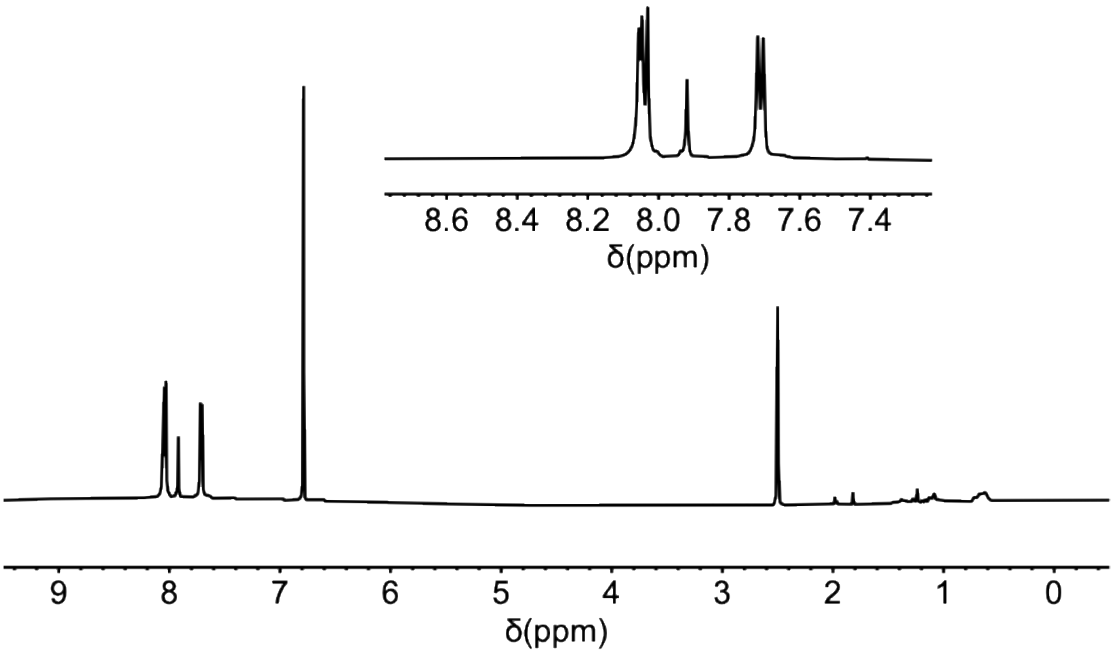

Figure S38: ^{1}H-NMR (d_6-DMSO, 500 MHz) of the crude product after photodegradation with NU-1000 (10 mg, 4.59 µmol), TEMPO (7.2 mg, 10 eq.), acetonitrile (2.5 mL), DI H_2O (1.25 µL), 33 °C, 390 nm, O_2, 18 h. TCE as internal standard at $\delta = 6.8$ ppm.

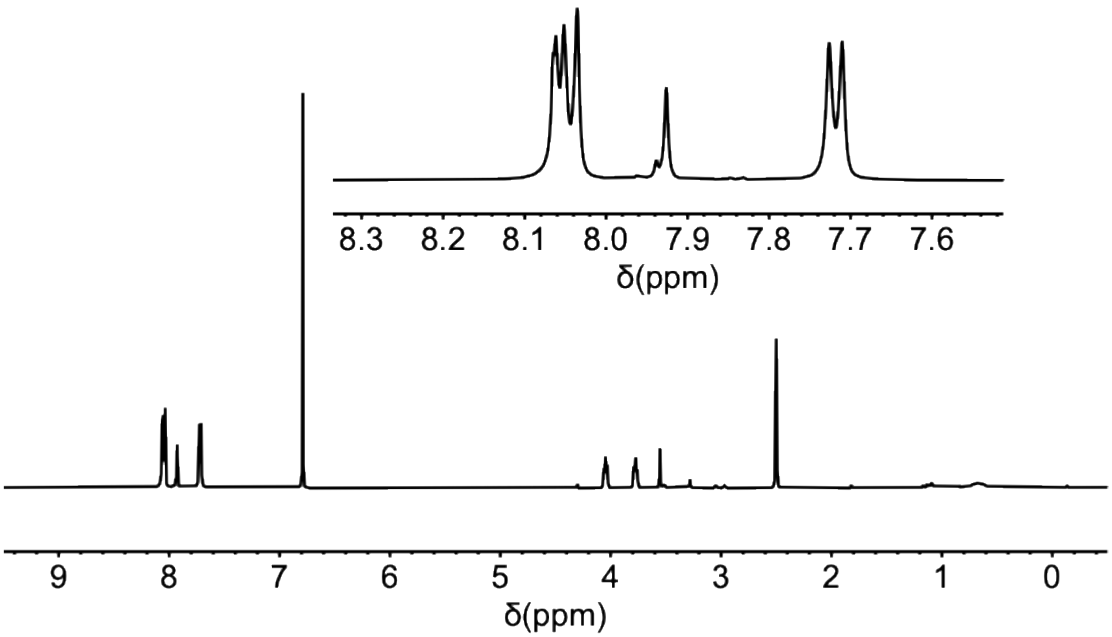

Figure S39: ^{1}H-NMR (d_6-DMSO, 500 MHz) of the crude product after photodegradation with NU-1000 (10 mg, 4.59 µmol), DABCO (5.1 mg, 10 eq.), acetonitrile (2.5 mL), DI H_2O (1.25 µL), 33 °C, 390 nm, O_2, 18 h. TCE as internal standard at δ = 6.8 ppm.

$$\text{Conversion of } H_2BDC = \frac{2 * 0.02}{2 * 0.02 + 1} = 4\%$$

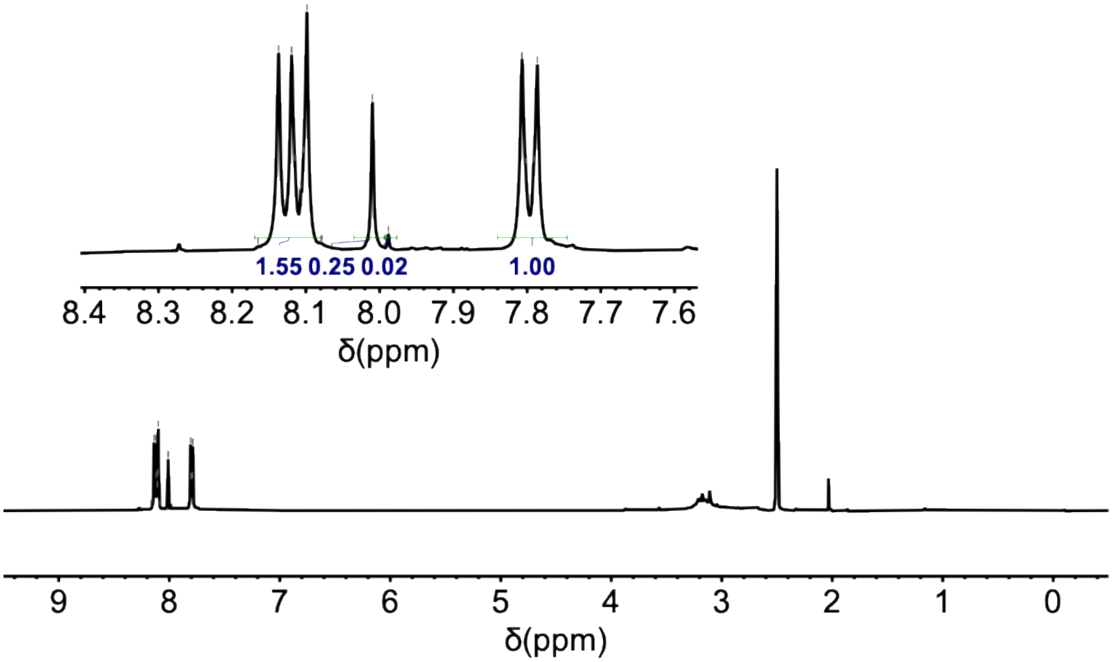

Figure S40: ^{1}H-NMR (d_6-DMSO, 500 MHz) of the crude product after photodegradation with NU-1000 (3.6 mg), TMPD (5 mg), MeCN (2.5 mL), DI H_2O (1.25 µL), 33 °C, 390 nm, O_2, 18 h.

$$\text{Conversion of } H_2BDC = \frac{2 * 0.39}{2 * 0.39 + 1} = 44\%$$

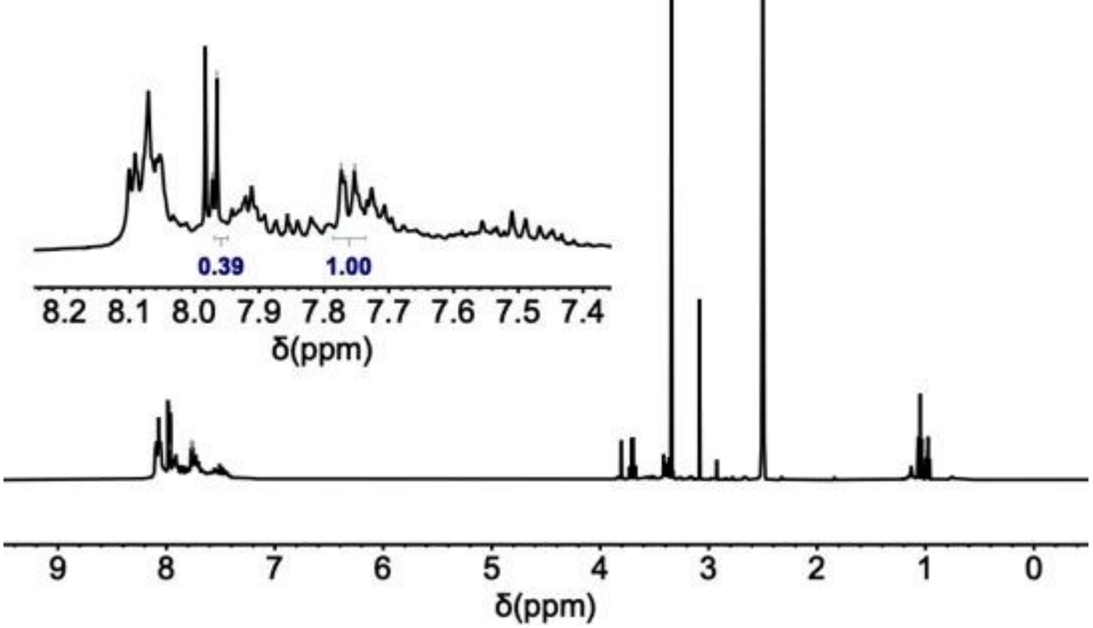

Figure S41: ^{1}H-NMR (d_6-DMSO, 500 MHz) of the crude product after photodegradation with NU-1000 (10 mg, 4.59 µmol), methanol (2.5 mL), DI H_2O (1.25 µL), 30 °C, 390 nm, O_2, 18 h.

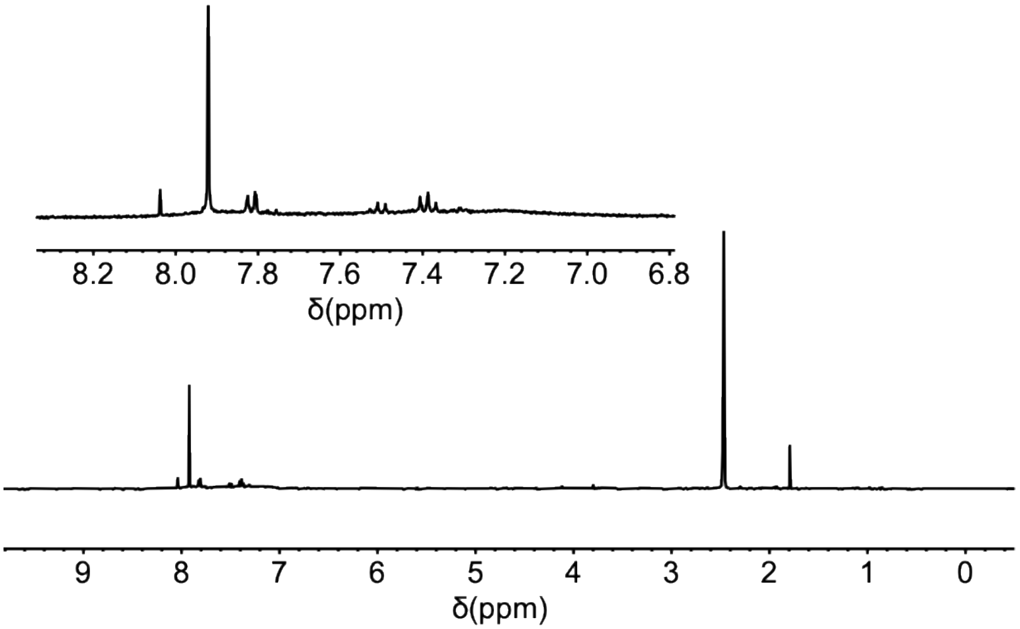

Figure S42: ^{1}H-NMR (d_6-DMSO, 500 MHz) of the crude product after photodegradation with styrene (21.5 µL, 0.188 mmol), NU-1000 (20 mg, 5 mol%), MeCN (5 mL), 33 °C, 390 nm, O_2, 18 h.

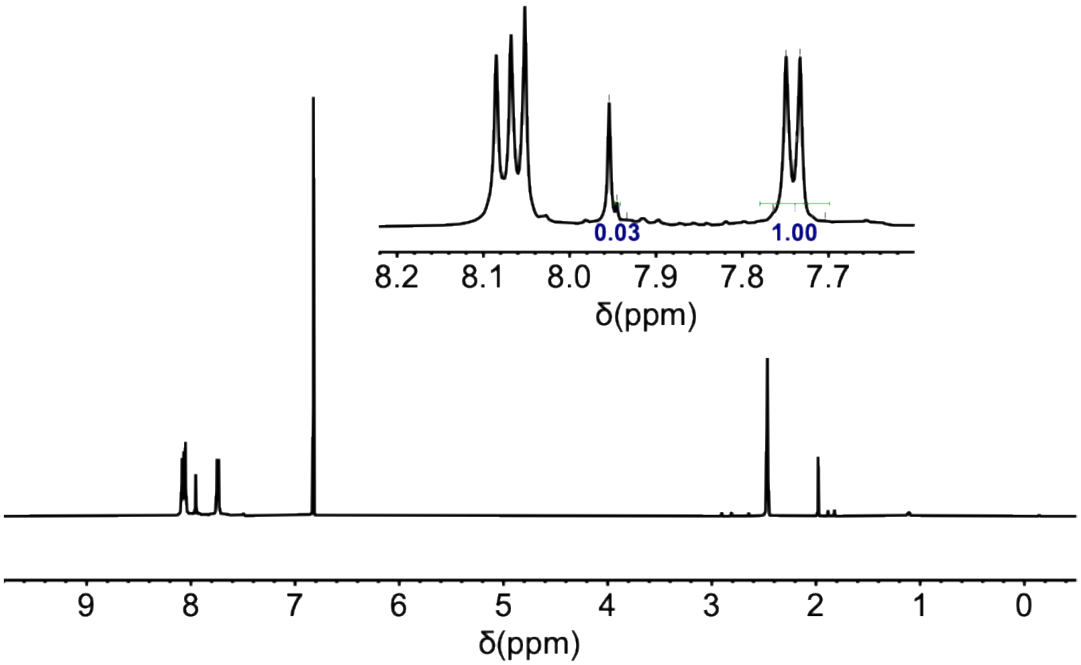

Figure S43: ^{1}H-NMR (DMSO-d_6, 500 MHz) of the crude product after photodegradation with NU-1000 (20 mg, 9.19 µmol), acetonitrile (5 mL), DI H_2O (2.5 µL), 33 °C, 390 nm (25% intensity), O_2, 18 h. TCE as internal standard at δ = 6.8 ppm.

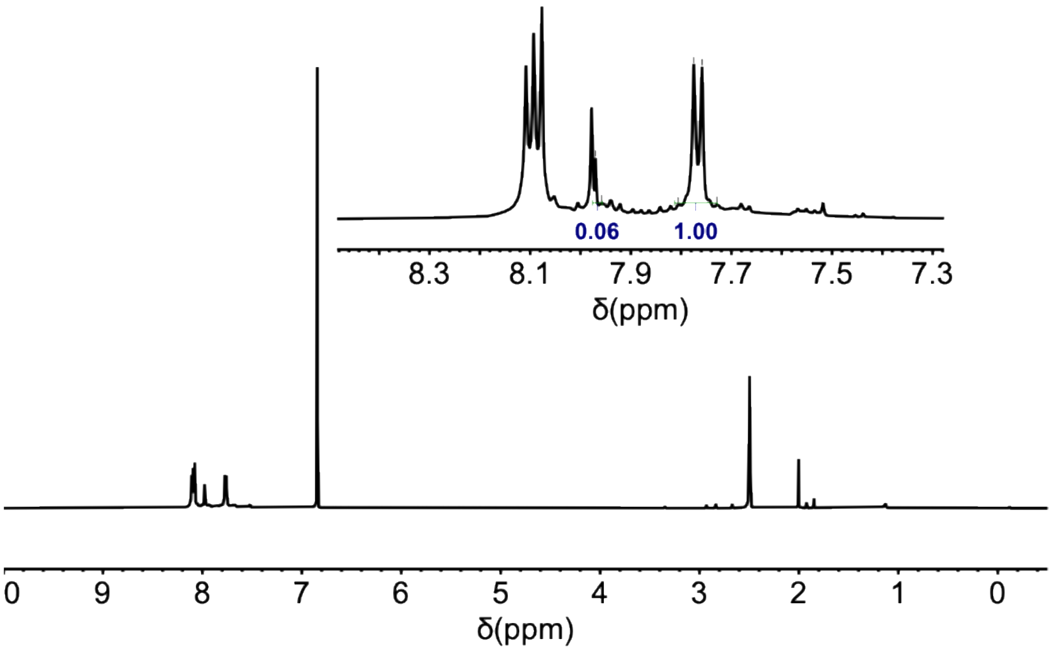

Figure S44: ^{1}H-NMR (DMSO-d_6, 500 MHz) of the crude product after photodegradation with NU-1000 (20 mg, 9.19 µmol), acetonitrile (5 mL), DI H_2O (2.5 µL), 33 °C, 390 nm (50% intensity), O_2, 18 h. TCE as internal standard at δ = 6.8 ppm.

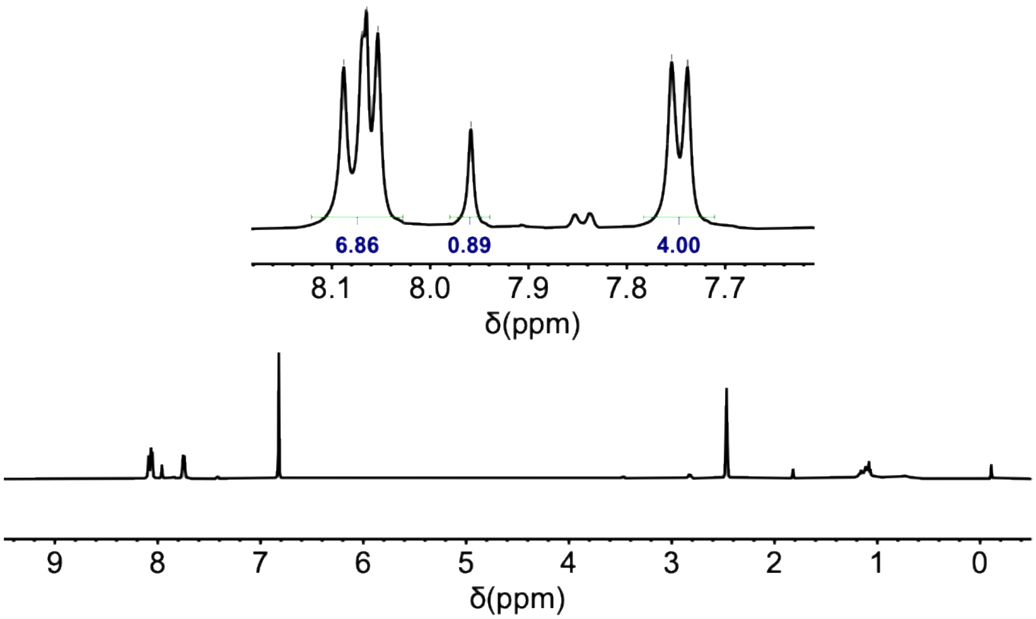

Figure S45: ^{1}H-NMR (d$_6$-DMSO, 500 MHz) of the crude product after photodegradation with NU-1000 (10 mg, 4.59 µmol), triethylamine (6.4 µL, 10 eq.), acetonitrile (2.5 mL), DI H_2O (1.25 µL), 33 °C, 390 nm, O_2, 18 h. TCE as internal standard at δ = 6.8 ppm.

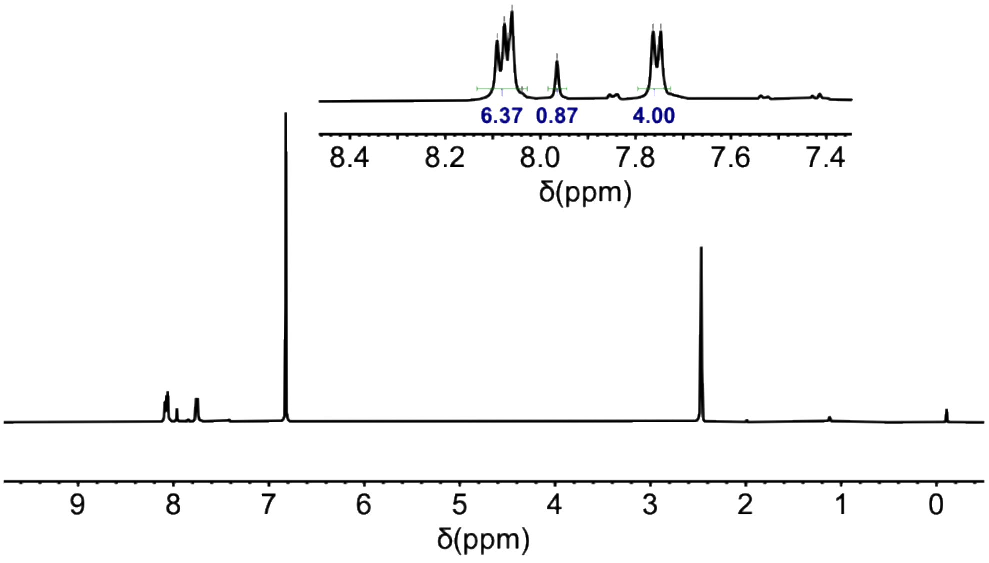

Figure S46: ^{1}H-NMR (d$_6$-DMSO, 500 MHz) of the crude product after photodegradation with NU-1000 (3.6 mg), 1,5-DHN (5 mg, 10 eq.), acetonitrile (2.5 mL), DI H_2O (1.25 µL), 33 °C, 390 nm, O_2, 18 h. TCE as internal standard at δ = 6.8 ppm.

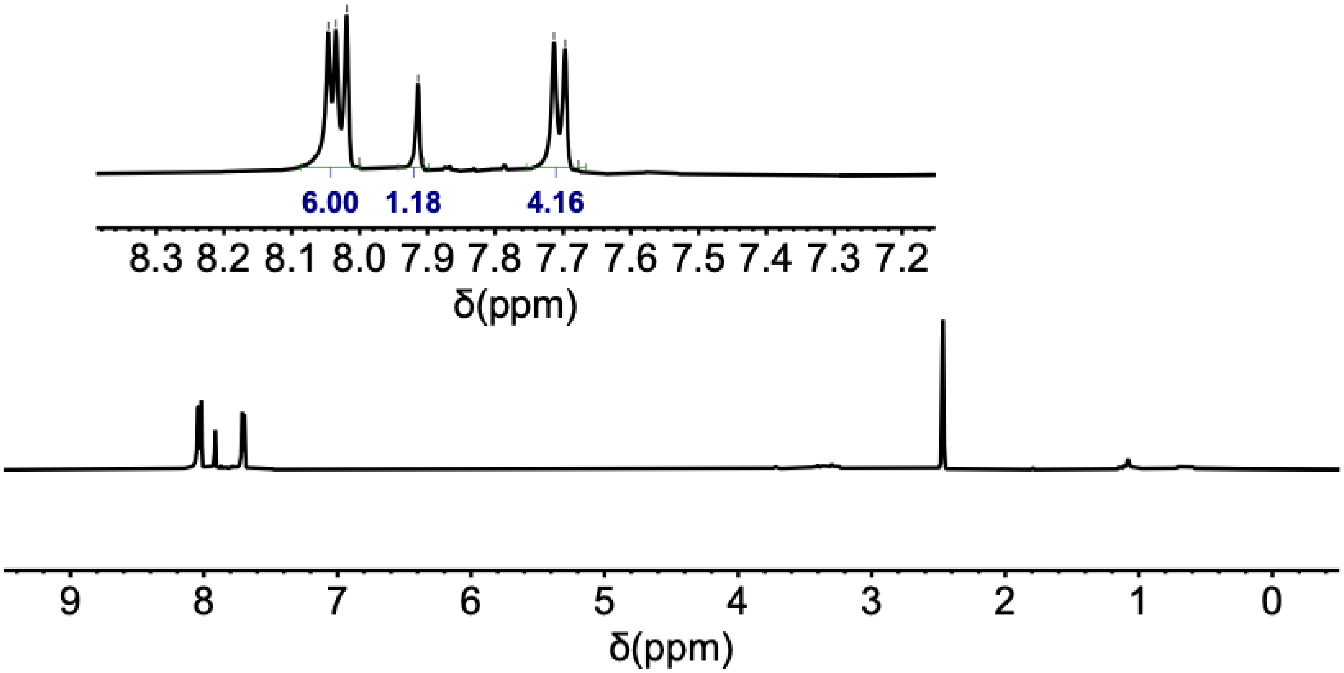

Figure S47: ^{1}H-NMR (d_6-DMSO, 500 MHz) of the crude product after photodegradation with NU-1000 (3.6 mg), HEPES (4 mg, 10 eq.), DI H_2O (2 mL), 33 °C, 390 nm, O_2, 18 h.

Reference

1. Wang, T. C.; Vermeulen, N. A.; Kim, I. S.; Martinson, A. B. F.; Stoddart, J. F.; Hupp, J. T.; Farha, O. K., Scalable synthesis and post-modification of a mesoporous metal-organic framework called NU-1000. *Nature Protocols* **2016,** *11* (1), 149-162.
2. Ong, A.; Wong, Z. C.; Chin, K. L. O.; Loh, W. W.; Chua, M. H.; Ang, S. J.; Lim, J. Y. C., Enhancing the photocatalytic upcycling of polystyrene to benzoic acid: a combined computational-experimental approach for acridinium catalyst design. *Chemical Science* **2024,** *15* (3), 1061-1067.
3. Garibay, S. J.; Iordanov, I.; Islamoglu, T.; DeCoste, J. B.; Farha, O. K., Synthesis and functionalization of phase-pure NU-901 for enhanced CO2 adsorption: the influence of a zirconium salt and modulator on the topology and phase purity. *CrystEngComm* **2018,** *20* (44), 7066-7070.
4. Ma, K.; Li, P.; Xin, J. H.; Chen, Y.; Chen, Z.; Goswami, S.; Liu, X.; Kato, S.; Chen, H.; Zhang, X.; Bai, J.; Wasson, M. C.; Maldonado, R. R.; Snurr, R. Q.; Farha, O. K., Ultrastable Mesoporous Hydrogen-Bonded Organic Framework-Based Fiber Composites toward Mustard Gas Detoxification. *Cell Rep. Phys. Sci.* **2020,** *1* (2), 100024.
5. Rigden, J. S., *Macmillan Encyclopedia of Physics*. Simon & Schuster Macmillan: 1996.
6. Kruse, O.; Rupprecht, J.; Mussgnug, J. H.; Dismukes, G. C.; Hankamer, B., Photosynthesis: a blueprint for solar energy capture and biohydrogen production technologies. *Photochemical & Photobiological Sciences* **2005,** *4* (12), 957-970.
